# Intracellular Binding of Novel Fluorinated Compounds to Carbonic Anhydrase Isoforms Explored by In-Cell ^19^F NMR

**DOI:** 10.1021/acs.jmedchem.5c02227

**Published:** 2025-10-27

**Authors:** Azzurra Costantino, Letizia Barbieri, Simone Giovannuzzi, Alessio Nocentini, Claudiu T. Supuran, Mindaugas Raitelaitis, Pär Nordlund, Lucia Banci, Enrico Luchinat

**Affiliations:** a CERM − Magnetic Resonance Center, 9300 University of Florence, via Luigi Sacconi 6, Sesto Fiorentino 50019, Italy; b 524266 Consorzio Interuniversitario Risonanze Magnetiche di Metallo Proteine − CIRMMP, via Luigi Sacconi 6, Sesto Fiorentino 50019, Italy; c NEUROFARBA Department, Section of Pharmaceutical and Nutraceutical Sciences, 9300 University of Florence, via Ugo Schiff 6, Sesto Fiorentino 50019, Italy; d Department of Oncology-Pathology, 27106 Karolinska Institutet, Stockholm 171 11, Sweden; e Chemistry Department, University of Florence, Via della Lastruccia 3, Sesto Fiorentino 50019, Italy

## Abstract

One of the most important challenges of drug development is identifying whether a candidate molecule can efficiently reach and engage intracellular targets. Cell-based assays are usually employed to address this issue; nevertheless, many compounds that appear promising in vitro fail in cellular or in vivo contexts due to low intracellular activity or off-target effects. In this work, we employed ligand-based in-cell ^19^F NMR to characterize ligand–target interactions in the cellular environment. We investigated the binding of newly synthesized fluorinated sulfonamide derivatives to various cytosolic isoforms of carbonic anhydrase in living human cells. We assessed target engagement and membrane permeability of each ligand and provided a qualitative affinity ranking of the compounds toward each isoform. The fluorinated compounds characterized in this study are promising scaffolds for the development of isoform-selective inhibitors and may serve as chemical probes for future competitive screenings of nonfluorinated drug candidates in cells.

## Graphical abstract

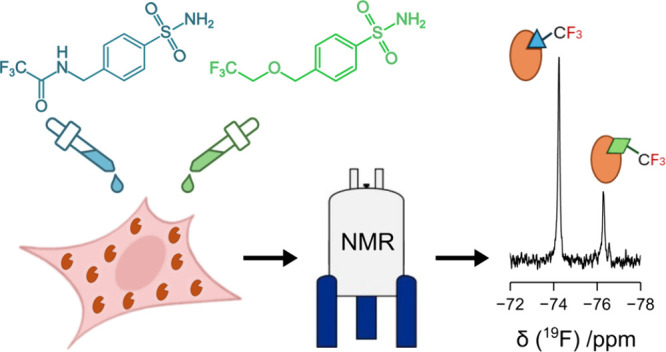

## Introduction

Drug design and implementation is a complex, multistep process in which candidate molecules undergo multiple steps of screening, selection, and optimization before they can reach the clinical trial phases. Starting from target identification, numerous ligand-based screening approaches can be used to identify compounds that interact with the target of interest, starting from various types of libraries. These screenings, integrated with protein-based structural studies to characterize the binding to the target protein, reduce the set of molecules, which could allow the rational design of lead compounds with increased binding affinity and possibly selectivity to the target. An essential further step is the assessment of the compound’s ability to reach intracellular targets, which is usually evaluated with ad hoc cell-based assays. Frequently, drug candidates that are effective in vitro fail on this aspect or at later stages in the cellular evaluation.^1,2^

Nuclear magnetic resonance (NMR) spectroscopy in solution is often employed to characterize lead compounds and screen for activity against their targets in vitro. Thanks to its nondestructive nature, NMR can also investigate intermolecular interactions, such as the binding of drug candidates to their target, directly in living cells.^3–5^ The in-cell NMR approach, applied to living cells overexpressing the target of interest and exposed to the candidate drugs, enables the assessment of target engagement in living human cells.^6,7^ The combination of in-cell NMR studies with a flow system further allows real-time monitoring of ligand binding, providing insights into the compound permeability by measuring membrane diffusion kinetics.^8–10^ In-cell NMR studies on ligand-target interactions can exploit both a target-based approach and a ligand-based approach. The target-based approach employs isotope-labeled intracellular targets and provides information on cell permeability and on the binding site location on the target protein.^6,8,11^ The ligand-based approach can provide information on cell permeability, target binding, and can also reveal off-target binding.^8,12^ Furthermore, by observing the ligand, it is possible to detect binding to target proteins difficult to observe directly by NMR due to interactions with the cellular environment. While ligand-based NMR in vitro relies on ^1^H spectra, in-cell detection requires additional means for selective observation of the molecule of interest. Recently, fluorine-19 (^19^F) NMR has shown its potential usefulness in structural biology and in drug development.^13,14^ ^19^F is 100% abundant, has high sensitivity (83% of ^1^H), and is naturally absent from biological systems. These characteristics make it a powerful tool for probing molecular interactions in living cells, while greatly reducing background interference.^14,15^ Indeed, in-cell ^19^F NMR has been applied to detect both intracellular proteins,^16–20^ nucleic acids,^21^ and small molecules carrying one or more fluorine atoms.^10,22,23^

In this study, we employed in-cell ^19^F NMR spectroscopy to characterize novel sulfonamide-based inhibitors of carbonic anhydrase (CA). CAs are zinc-containing enzymes found across all domains of life that catalyze the reversible hydration of carbon dioxide to bicarbonate. This reaction plays a crucial role in several physiological processes, including pH regulation, CO_2_ transport, and respiration. In humans, 15 α-class CA isoforms are known, each with distinct tissue distribution and subcellular localization. Cytosolic isoforms such as CA1, CA2, and CA3 are highly expressed in erythrocytes, muscle, and various organs, while mitochondrial isoform CA5 is localized in the kidney, liver, and pancreas, where it contributes to biosynthetic pathways. Membrane-bound isoforms such as CA9 and CA12, frequently overexpressed in tumors, are found in the gastrointestinal tract and reproductive tissues, whereas the secreted isoform CA6 is present in saliva. Two additional cytosolic isoforms are expressed in the central nervous system (CA7) and in the small intestine, prostate, and colon (CA13).^24,25^ Their functional diversity and isoform-specific tissue distribution make CAs valuable therapeutic targets. Several CA inhibitors are already used clinically, while others are currently in development for several therapeutic applications, where achieving isoform-selective inhibition is critical.^24–26^ The compounds reported here were specifically designed for in-cell detection; thereby, they incorporate one or more trifluoromethyl groups (−CF_3_) to enable detection with high sensitivity via ^19^F NMR. The binding of these compounds to a panel of cytosolic CA isoforms (CA1, CA2, CA3, CA7, and CA13) was assayed in human cells by in-cell ^19^F NMR. These cytosolic CA isoforms share a highly conserved catalytic domain, with the Zn^2+^ coordinated by His94, His96, and His119, and a key proton shuttle involving His64. CA2, the most ubiquitous and extensively studied isoform, along with CA1, CA7, and CA13, displays high catalytic activity. In contrast, CA3, primarily expressed in skeletal muscle and its precursors, exhibits a significantly reduced enzymatic efficiency due to a lysine substitution at the His64 position.^27,28^ To explore selective binding across these highly similar cytosolic isoforms, we established a qualitative affinity ranking of the compounds for each CA isoform and assessed their cellular uptake kinetics. Additionally, we applied the cellular thermal shift assay (CETSA) to independently confirm the target engagement of the compounds to endogenously expressed CA2. These compounds could be used as scaffolds for the development of novel isoform-selective CA inhibitors. Furthermore, one of the compounds, which shows markedly faster membrane diffusion kinetics, will be a promising chemical probe for screening nonfluorinated CA inhibitors via competition binding in-cell ^19^F NMR.^10^

## Results and Discussion

The expression level of five different human cytosolic CA isoforms—CA1, CA2, CA3, CA7, and CA13—in transiently transfected cultured human cells was evaluated. CA1, CA2, and CA3 were significantly overexpressed, reaching average intracellular concentrations between 130 and 170 μM, whereas CA7 and CA13 were expressed at lower levels, with concentrations of ∼50 and ∼80 μM, respectively, but still within the sensitivity range of in-cell NMR experiments (Figure S1). In principle, such target concentrations are compatible with target-based in-cell NMR ligand binding studies.^6^ However, slow tumbling caused by interactions with cellular components may hamper NMR detection of the target proteins. The intracellular behavior of each isoform was therefore evaluated by in-cell^1^H–^15^N NMR. CA1, CA2, and CA3 resulted in well-resolved signals, whereas no signals were detected for CA7 and CA13, except for those arising from the highly dynamic FLAG tag (Figure 1). All isoforms were clearly detected in the NMR spectra of the corresponding cell lysates, confirming that in intact cells, CA7 and CA13 experience line broadening beyond detection due to intracellular interactions.

**Figure 1:**
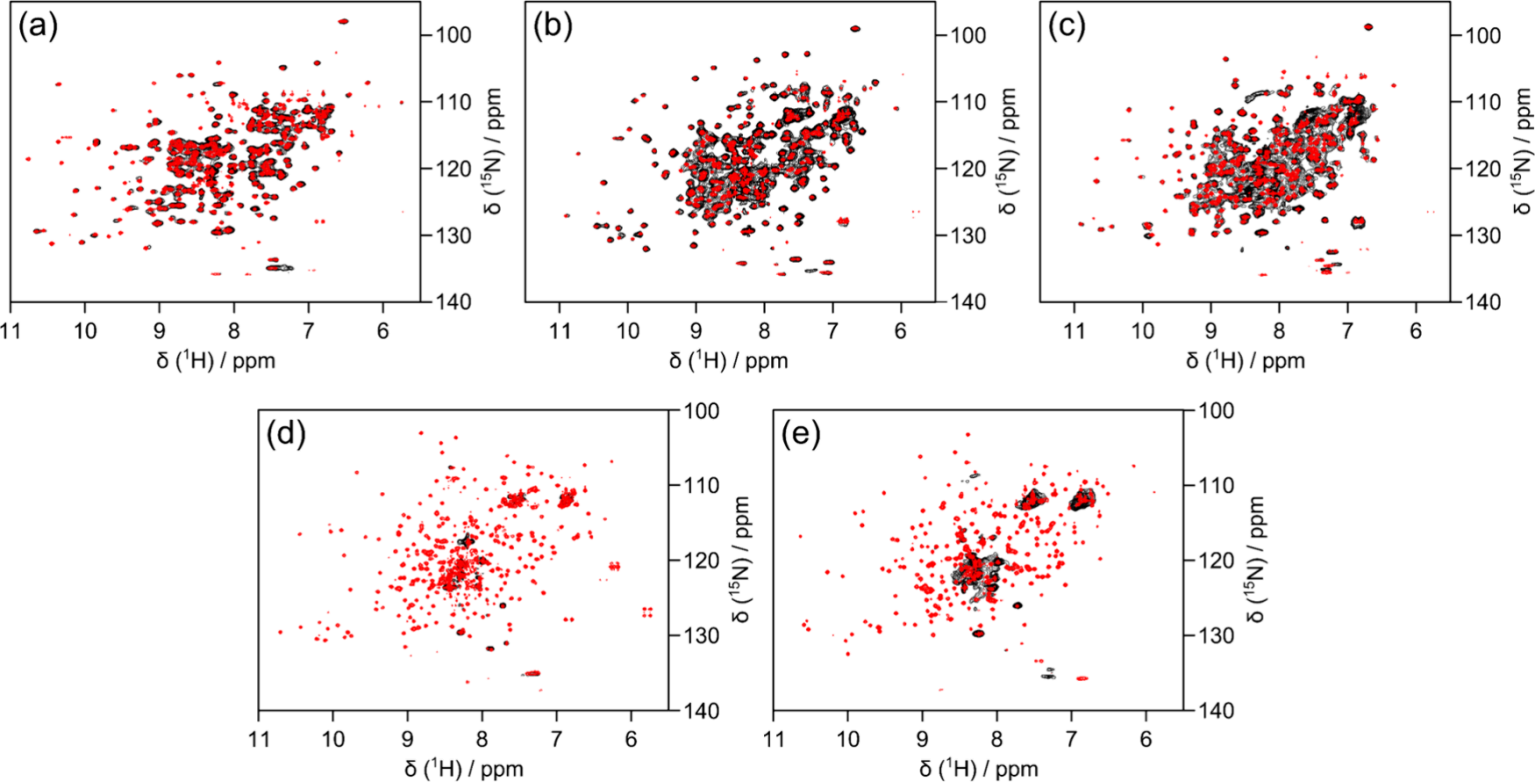
^1^H–^15^N NMR spectra of CA isoforms. 2D ^1^H–^15^N BEST-TROSY in-cell NMR spectra (black) and cell lysate NMR spectra (red) of CA1 (a), CA2 (b), CA3 (c), CA7 (d), and CA13 (e).

In contrast to target-based approaches based on the detection of the protein backbone (e.g., by ^1^H–^15^N 2D spectra), ligand-observed in-cell ^19^F NMR experiments are less crowded and less affected by the tumbling rate of the complex, allowing ligand screening studies also for slow-tumbling targets.^10^ Furthermore, the binding of multiple ligands can be investigated simultaneously thanks to the high sensitivity of the ^19^F chemical shift to the local environment.

For this study, a novel series of fluorinated benzenesulfonamides (compounds **1**–**7**, Chart 1) was designed as potential ligands for different CA isoforms. In these compounds, a trifluoromethyl group (−CF_3_) was incorporated onto the substituents of the benzenesulfonamide moiety. Sulfanilamide and SLC-0111, two established and potent carbonic anhydrase inhibitors (CAIs), were the lead structures for the design of the fluorinated ligands. Compound **1** is a derivative of sulfanilamide previously reported as a CAI,^29^ while compounds **2** and **3** are congeners of compound **1**, distinguished by the presence of extended linkers between the CAI core and the −CF_3_ group (Schemes 1 and 2). Compounds **4** (reported previously^30^) and **5** (Scheme 3) are analogues of SLC-0111, incorporating a −CF_3_ or −OCF3 group in place of a single fluorine atom. Compound **6** represents an analogue of compound **2** that features an ether linker, as opposed to the amide found in its predecessor. Compound **7** is structurally related to compound **6** and is characterized by the inclusion of additional −CF_3_ groups on its terminal segment (Scheme 4). This focused structural diversification aimed to explore the impact of linker type and degree of fluorination on carbonic anhydrase inhibition. Compared to other groups containing nonequivalent fluorine atoms, the −CF_3_ provides a 3-fold increase in NMR signal intensity and favorable relaxation properties. This property was maximized in compound **7**, which contains three equivalent CF_3_ groups.

**Chart 1:**
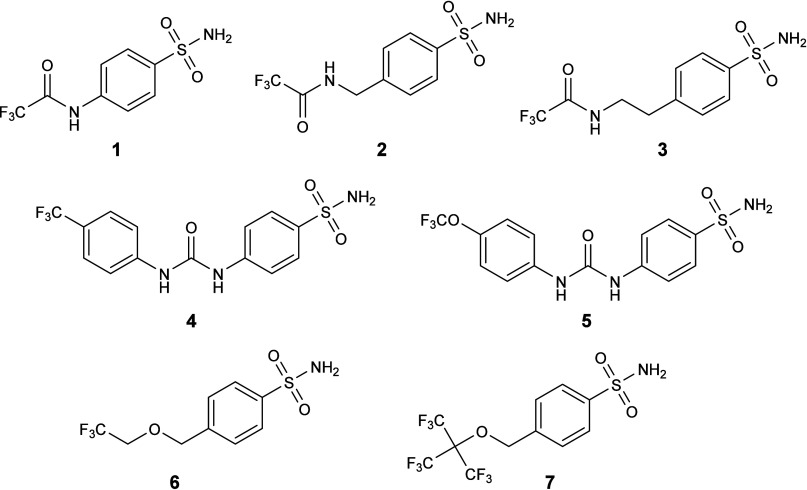
Structures of the Fluorinated Benzenesulfonamide Derivatives Tested in This Study

**Scheme 1:**
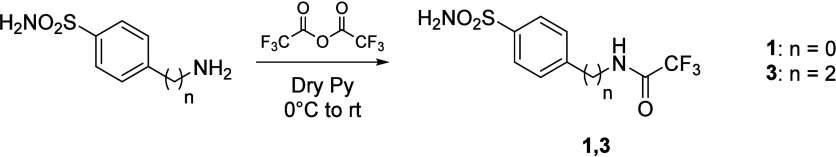
Synthesis of Compounds **1** and **3**

**Scheme 2:**
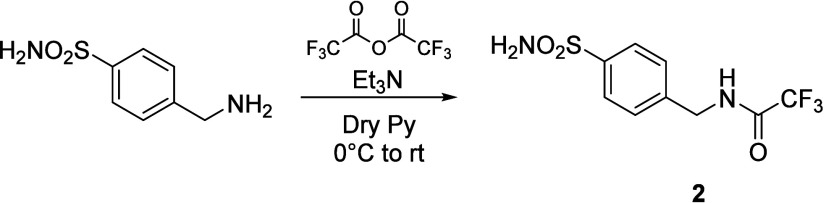
Synthesis of Compound **2**

**Scheme 3:**
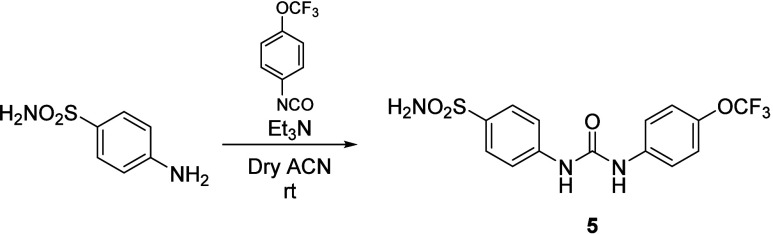
Synthesis of Compound **5**

**Scheme 4:**
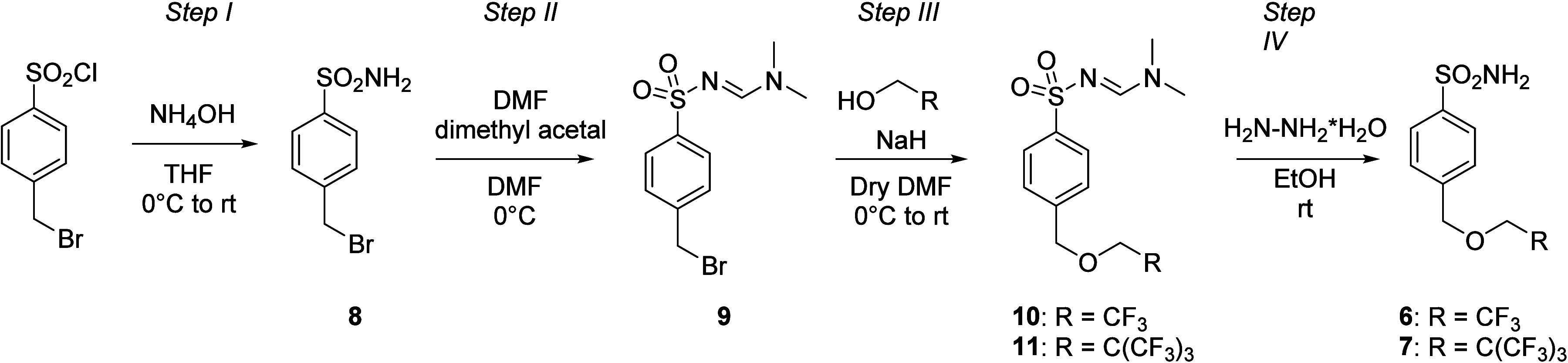
Synthesis of Compounds **6** and **7**

The inhibition constants (*K*_I_s) of the compounds against each cytosolic CA isoform were evaluated in vitro through a stopped-flow assay (Table 1) and compared to that of the reference compound acetazolamide (AAZ), a clinically established CA inhibitor. All the compounds exhibited inhibitory activity against CA1, CA2, CA7, and CA13, with comparable K_I_ values, while none of the compounds inhibited the CA3 isoform (*K*_I_ > 100 μM), including AAZ. This is consistent with the known resistance of CA3 to benzenesulfonamide-based ligands, which arises from the substitution of a leucine with phenylalanine in the active site that makes the CA3 ligand binding pocket inaccessible to aromatic sulfonamides.^31^

**Table 1:** In Vitro Inhibition Data (*K*_I_ Values in nM) of Human Cytosolic CA Isoforms by Compounds **1**–**7** and the Reference Drug Acetazolamide (AAZ)

	***K***_**I**_ **(nM)** ^a^				
**compound**	**CA1**	**CA2**	**CA3**	**CA7**	**CA13**
**1**	6550	145	>100 μM	178	90.2
**2**	1260	90.9	>100 μM	107	146
**3**	988	121	>100 μM	76.9	55.8
**4**	9.7^b^	1150^b^	>100 μM	552	153
**5**	142	203	>100 μM	289	82.0
**6**	49.3	78.0	>100 μM	101	230
**7**	81.6	57.6	>100 μM	69.3	124
AAZ	250.0	12.0	>100 μM	5.7	17.0

a Mean from three different assays, by a stopped-flow technique (errors were in the range of ±5–10% of the reported values).

b Taken from Pacchiano et al.^30^

The stability of the fluorinated ligands in the cell culture medium was assessed through ^19^F NMR (Figure S2). Compounds **2**–**7** were stable for one hour at 37 °C, while compound **1** showed the appearance of an additional signal over time, with the simultaneous decrease of the initial signal, suggesting a spontaneous hydrolysis of the trifluoroacetamide moiety. The hydrolysis of compound **1** in water and the consequent formation of sulfanilamide were confirmed by LC-MS (Figures S3–S5).

The binding of the compounds to each cytosolic CA isoform was then analyzed by in-cell ^19^F NMR spectroscopy. Incubating the cells for 1 h with an excess of each compound resulted in the formation of the intracellular complex with CA1, CA2, CA7, and CA13, giving rise to a signal characteristic for each isoform (Figure 2). These signals were absent in control samples of nonoverexpressing cells treated with the same compounds, confirming that they arise from the binding to the targets. Notably, compound **1** also formed a stable complex with intracellular CA isoforms, despite being gradually hydrolyzed in the external medium during the incubation phase. As expected, no complex formation occurred in the cells expressing CA3, consistent with the lack of inhibition observed through the stopped-flow assay. An additional signal, weaker but sharper, was also observed in some of the spectra. The same signal is also present in the ^19^F NMR spectra of the supernatant fractions from each cell sample, and its chemical shift corresponds to that of the free compound, thus suggesting that a small fraction of the compound is released from the cells during the in-cell NMR analysis (Figure S6). The ^19^F NMR spectra of the cell lysates showed clear signals at the same chemical shifts observed in the intact cells, indicating that the complexes formed in the cells were preserved after cell lysis (Figure S7).

**Figure 2:**
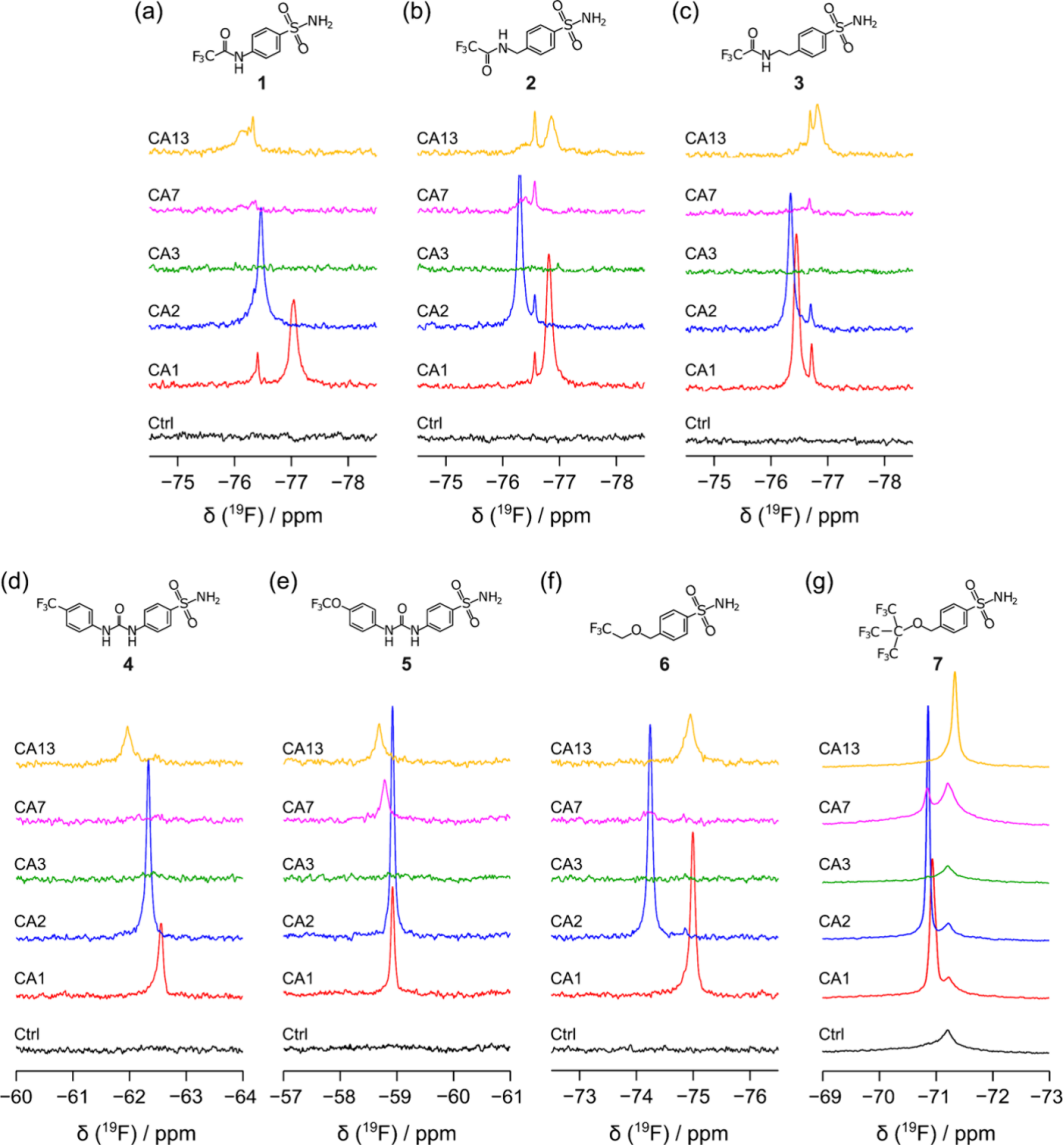
Binding of the fluorinated compounds to intracellular CA isoforms. 1D in-cell ^19^F NMR spectra of cells expressing different CA isoforms treated for 1 h with 100 μM of compound **1** (a), **2** (b), **3** (c), **4** (d), **5** (e), **6** (f), and **7** (g). The spectra are color-coded based on the CA isoform: CA1 (red), CA2 (blue), CA3 (green), CA7 (purple), and CA13 (yellow). The control spectra (black) of cells transfected with an empty vector are also shown.

While the spectra of control cells treated with compounds **1**, **2**, **3**, **4**, and **6** did not contain any signal (Figure 2a–d,f), compounds **5** and **7** gave rise to broad signals, which were also present in the CA-expressing cells, indicating that these compounds bind nonspecifically to other cellular components (Figure 2e, g and Figure S8). This suggests that compounds **5** and **7** may not be suitable for therapeutic targeting of CA isoforms due to their lack of specificity.

Based on the nano- to micromolar *K*_I_ values determined in vitro, all the compounds are expected to saturate the intracellular target proteins. In these experimental conditions, the signal intensities in the NMR spectra are determined by the level of target protein in each sample and do not report on the intracellular binding affinity of the compounds. Therefore, to determine the relative binding affinities of the fluorinated compounds for each cytosolic CA isoform, we performed intracellular competition binding experiments. To this aim, cells expressing CA1, CA2, and CA13 were treated for 1 h with two compounds simultaneously, followed by in-cell ^19^F NMR analysis as above. CA3 and CA7 were excluded from this analysis due to a lack of binding and low expression levels, respectively. Compounds **2**, **4**, **5**, **6**, and **7** were selected, while compound **1** was excluded due to its low stability in the culture medium, and compound **3** was omitted due to its structural similarity with compound **2**. The resulting spectra showed two distinct signals with different intensities, corresponding to the two compounds in complex with intracellular CA (Figure 3). Assuming that the incubation phase is sufficiently long to allow both compounds to reach the intracellular target, we reached a dynamic equilibrium such that the ratio of the signal intensities (normalized by the number of equivalent fluorine atoms) is directly proportional to the ratio of the binding affinities of the two compounds. This allowed us to establish a qualitative affinity ranking for each CA isoform. Notably, the affinity ranking profiles varied across the CA isoforms. Specifically, compounds **2** and **6** had lower affinity for CA2 and CA13 with respect to **5** and **4**, whereas the opposite was observed for CA1, while compound **7** showed the highest affinity for all three CA isoforms. Strikingly, the in-cell rankings did not correlate with the K_I_s measured in vitro. For example, compound **4** ranked the highest in vitro and was the second weakest in cells against CA1, while against CA2, it had the opposite behavior. These results suggest that the cellular context affects each ligand-target interaction differently in complex ways.

**Figure 3:**
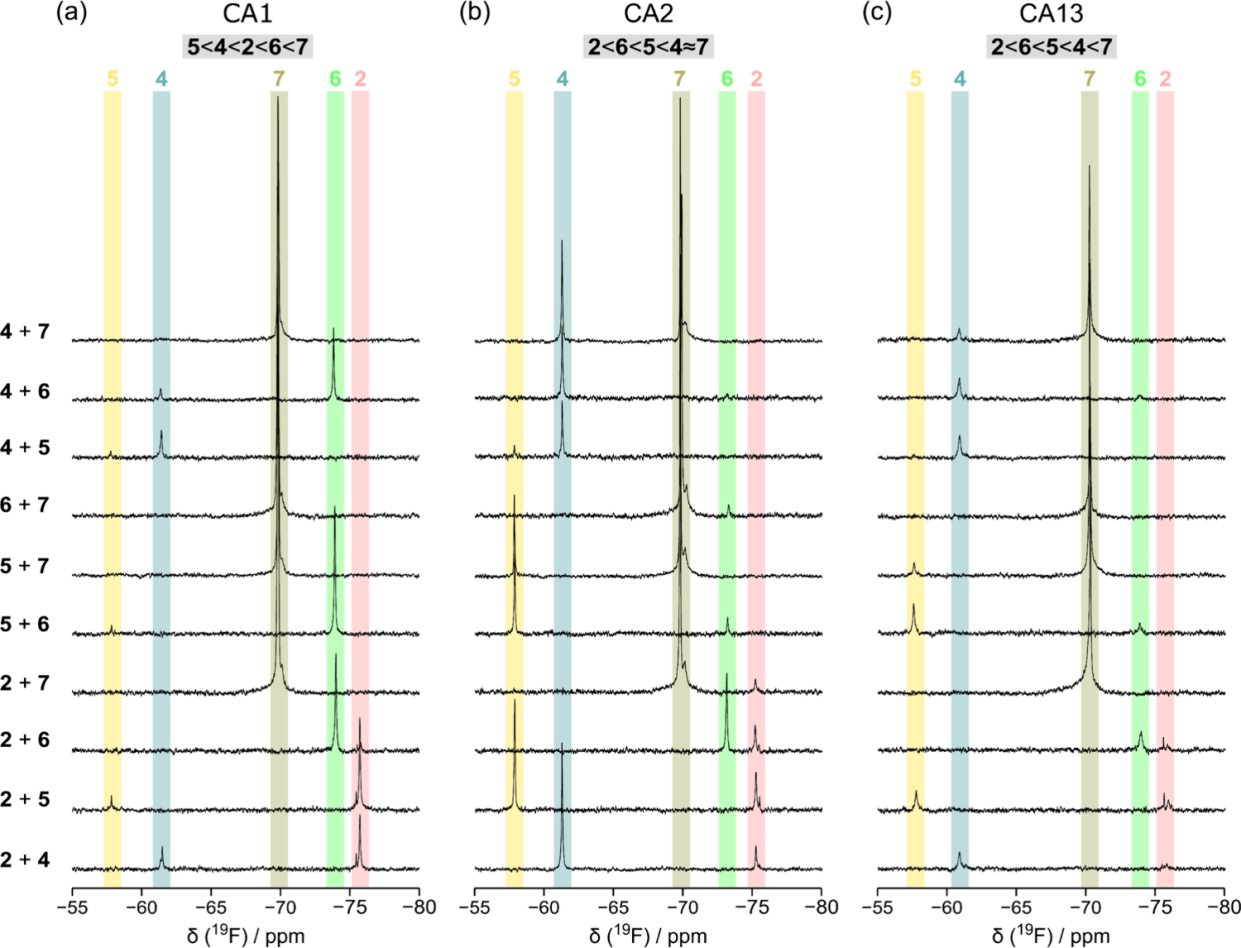
Competition binding and affinity ranking of the compounds to intracellular CA isoforms. 1D in-cell ^19^F NMR spectra of cells expressing different CA isoforms: CA1 (a), CA2 (b), and CA13 (c), treated with different pairs of fluorinated compounds (indicated on the left of each spectrum), 50 μM each for 1 h. The qualitative affinity rankings are reported at the top of each panel.

When recorded in real time using an NMR flow bioreactor,^32^ in-cell ^19^F NMR allows evaluating the cell permeability of fluorinated compounds. To this aim, cells overexpressing CA2 were encapsulated in agarose gel and perfused in the NMR bioreactor with a constant flow of medium containing 5 μM each ligand, while ^19^F NMR spectra were continuously recorded to measure the rate of intracellular complex formation. As the cellular uptake is the slow step in the ligand binding kinetics, the rate of complex formation is dominated by the membrane permeability, rather than the association rate constant to the intracellular target.^6^ Linear fitting of the fluorine signal intensity over time revealed that compound 6 had a markedly faster cellular uptake, whereas the other compounds had similar uptake rates (Figure 4).

**Figure 4:**
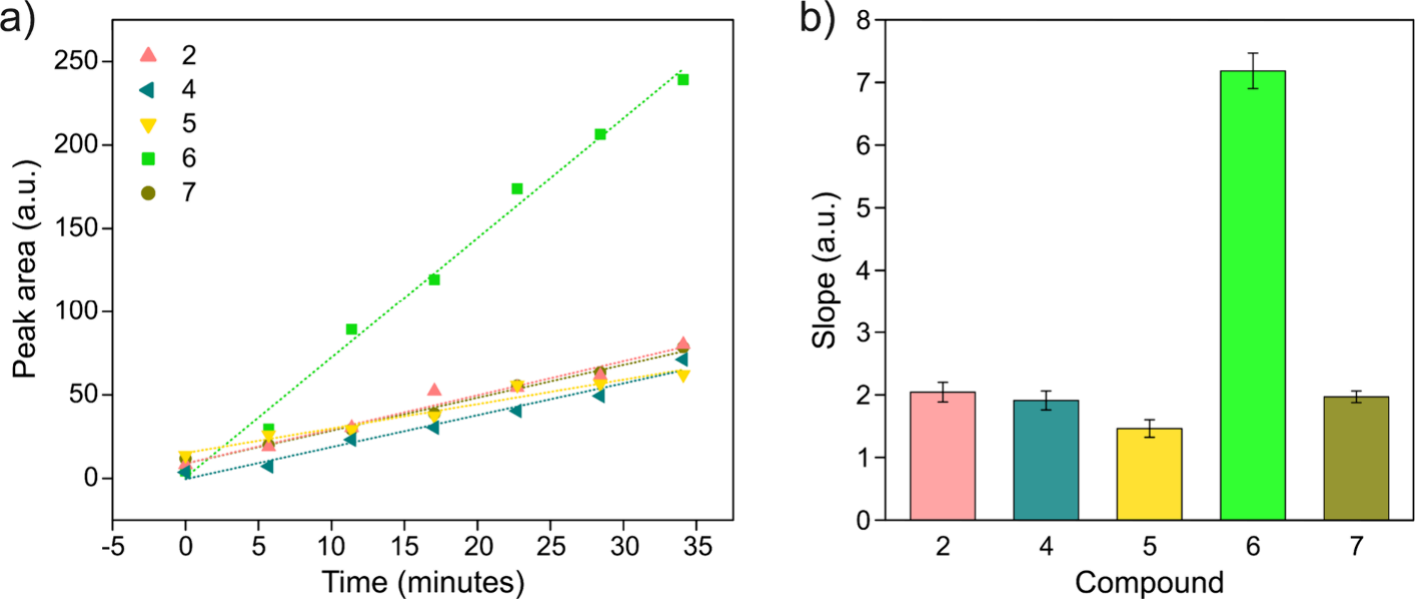
Uptake kinetics of fluorinated compounds in HEK293T cells expressing CA2. (a) Time-dependent ^19^F NMR signal integrals for each compound, acquired in real time by using the NMR bioreactor. (b) Histogram of the relative slopes obtained from fitting the fluorine signal integrals as a function of time, indicating the comparative uptake rates of the compounds.

Finally, CETSA experiments were performed to independently confirm the interaction between the fluorinated compounds and the endogenous CA2. CETSA is based on the principle that ligand binding stabilizes proteins against thermal denaturation in living cells.^33,34^ The assay was performed on compounds **2** and **4**–**7** ranked above (Figure 5). In cells treated with compounds **4**–**7**, the melting curves of endogenous CA2 showed melting temperatures (*T*_m_) ranging from ∼53 to ∼63 °C, which are noticeably higher than the *T*_m_ of unbound CA2 (50–52 °C). These findings are consistent with the in-cell ^19^F NMR spectra, further confirming that binding also occurred with endogenous protein levels. Interestingly, even though a protein-bound signal appeared in the in-cell ^19^F NMR spectrum, the melting curve for cells treated with compound **2** differed only slightly from the control (Figure 5a). This suggests that although compound **2** binds to CA2, as clearly seen by NMR, the interaction does not increase the thermal stability of the protein to a large extent. As our ligand-based NMR approach gives a strong signal, this highlights the complementarity of CETSA and in-cell NMR. While in the case of CETSA the protein reports on the interaction by being stabilized by the ligand, NMR provides a direct indication of the interaction regardless of the thermal stability of the resulting complex.

**Figure 5:**
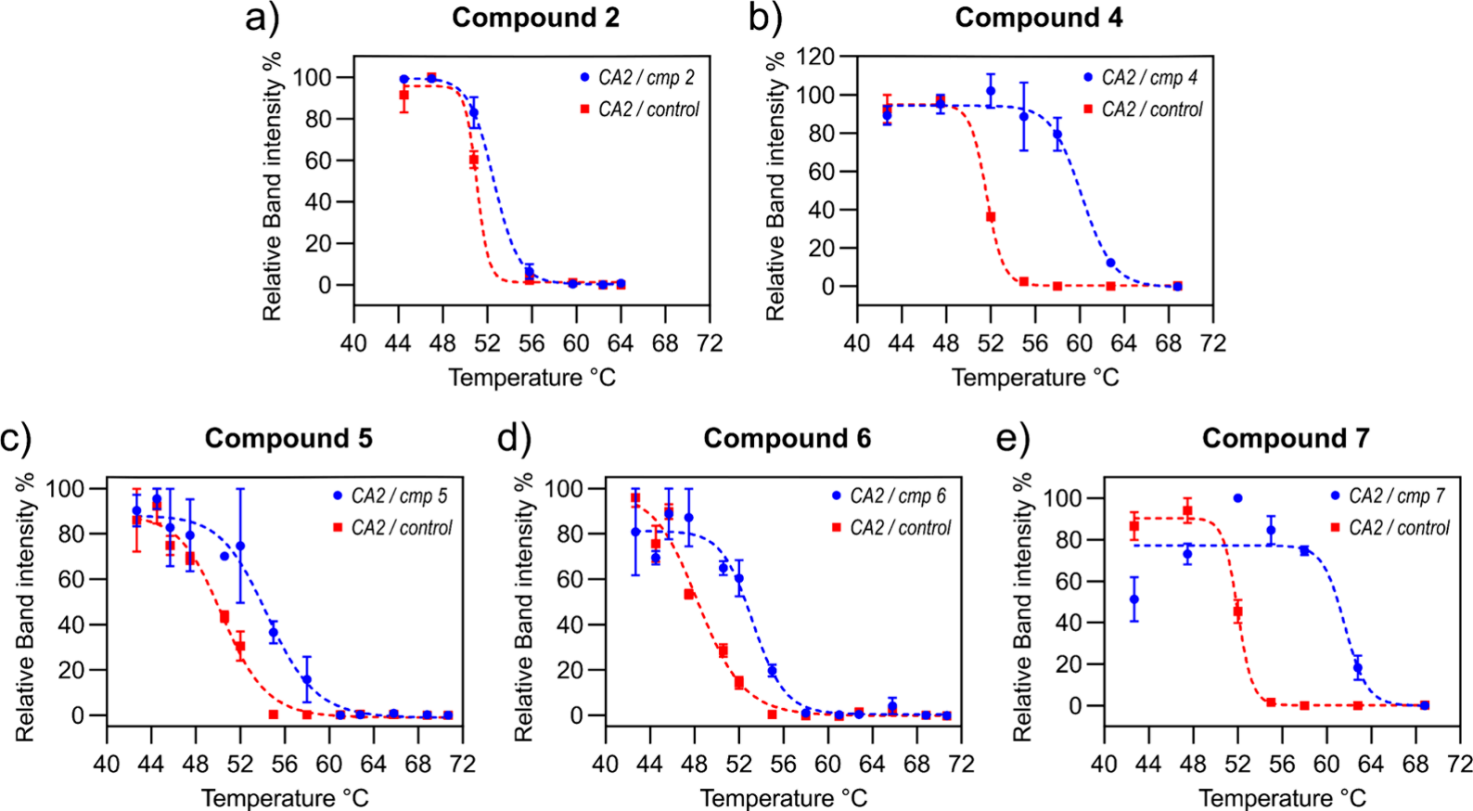
CETSA thermal stability curves of endogenous CA2 in cells treated with fluorinated compounds. CETSA profiles show the thermal stability of CA2 in cells treated with fluorinated compounds (blue curves) compared with DMSO-treated controls (red curves). Band intensities from Western blots using an anti-CA2 antibody were quantified and plotted against the temperature at which the cells were heated. Each panel corresponds to a different fluorinated compound used for treatment: (a) compound **2**, (b) compound **4**, (c) compound **5**, (d) compound **6**, and (e) compound **7**.

## Conclusions

In this work, we characterized the binding of newly synthesized fluorinated benzenesulfonamide-based inhibitors targeting cytosolic CA isoforms using in-cell ^19^F NMR spectroscopy. By employing this ligand-based approach, protein–ligand interactions were observed directly in the cellular environment, providing a physiologically relevant binding assay compared to classical in vitro binding studies. Among the five CA isoforms analyzed, CA1, CA2, CA7, and CA13 clearly exhibited binding interactions with compounds, whereas CA3 displayed no interaction, as expected due to the increased steric hindrance in its active site. In the intracellular affinity ranking, compound **7** consistently showed the highest affinity among the isoforms; however, along with compound **5**, it also displayed additional NMR signals likely arising from nonspecific interactions, which may be detrimental for future therapeutic applications and should be investigated further. The in-cell affinity rankings were significantly different from those based on enzyme inhibition in vitro, further highlighting the importance of performing early-stage screenings in a cellular model, as doing so will affect the choice of promising lead compounds, with important consequences for any further drug development. Uptake kinetics analysis by real-time NMR revealed that compound **6** has higher membrane permeability compared to the others, making it a promising hit compound for future drug development, and the best candidate as a “spy” ligand for affinity ranking studies of other hit/lead compounds based on competition binding and ^19^F NMR detection. CETSA assays revealed ligand-induced thermal stabilization of native CA2, which further validates the binding data acquired through in-cell NMR. Although compound **2**, which showed binding in NMR spectra, only showed a weak thermal stabilization of the protein, compounds **4**–**7** increased the melting temperature of CA2, confirming binding to endogenous protein levels. This underscores the complementarity between CETSA and NMR: the former is more sensitive and can also be applied to the endogenous protein, while the latter provides direct evidence of the interaction regardless of thermal stability and provides further relevant data from affinity rankings and uptake kinetics. The fluorinated ligands developed in this work represent promising scaffolds for further optimization and development of specific CA inhibitors. Moreover, thanks to their fluorine content, some of these compounds may also be used as probes for competitive screening of nonfluorinated candidates in cellular environments.

Finally, from a methodological point of view, this approach overcomes some of the limitations of target-based in-cell NMR methods, in which some targets may not be detected due to interactions with cellular components, causing extensive broadening of the protein NMR signals. The strategy employed here allows a relatively fast ranking of the compounds based on their binding affinity in cells. However, this ranking is to be taken qualitatively, as several factors can affect the intracellular binding equilibrium, such as the release of ligand from the cells during the analysis or additional nonspecific interactions with cellular components. Performing the same experiments in an NMR bioreactor would allow higher control of the ligand concentrations and of the sample conditions, enabling a quantitative affinity ranking purely based on the intracellular *K*_d_s. In general, these results highlight the potential of in-cell ^19^F NMR as a powerful and versatile technique for directly investigating ligand–target interactions in living cells.

## Experimental Section

### Gene Constructs

Codon-optimized sequences encoding different cytosolic carbonic anhydrase isoforms (CA1, NP_001729; CA2, NP_000058.1; CA3, NP_005172.1; CA7, NP_001014435.1; CA13, NP_940986.1), with the secretion sequence removed as previously described,^6^ were obtained by gene synthesis and cloned into the pHLsec mammalian expression vector^35^ (Twist Bioscience). A FLAG tag (GSSDYKDDDDK) was added to the C-terminus of each CA isoform.

### Protein Expression in Human Cells

The HEK293T (ATCC CRL-3216) cell line was used for transient transfection, following a protocol described previously.^36^ Cells were cultured in a T75 flask using high-glucose Dulbecco’s modified Eagle medium (DMEM, Gibco), supplemented with 1% penicillin–streptomycin (Gibco) and 10% fetal bovine serum (Gibco), and maintained at 37 °C in a humidified incubator with 5% CO_2_. The cells were transiently transfected using polyethylenimine (PEI, Sigma-Aldrich) at a 1:2 ratio (25 μg DNA:50 μg PEI).^36^ After transfection, protein expression was carried out for 48 h in DMEM supplemented with 100 μg/mL of penicillin–streptomycin (Life Technologies), 2% (v/v) of fetal bovine serum (FBS, Life Technologies), and 10 μM of ZnSO_4_.

### Protein Quantification via SDS-PAGE

The expression levels of cytosolic CA isoforms were assessed by using SDS-polyacrylamide gel electrophoresis (SDS-PAGE). Transfected cells from T25 flasks were lysed in 150 μL of phosphate-buffered saline (PBS), and the soluble lysates were collected. As a negative control, a cell lysate was prepared from cells transfected with the same procedure but with a plasmid lacking the gene of interest. Each cell lysate sample was loaded at three different dilutions for quantification. To generate a linear regression standard curve for quantification, purified CA2 protein was loaded at different concentrations (20, 15, 10, 5, and 2.5 μM). Both samples and standards were mixed 2:1 with SDS loading buffer [300 mM Tris-HCl (pH 6.8), 6% (w/v) SDS, 30% (v/v) glycerol, 0.3% (w/v) bromophenol blue, and 150 mM dithiothreitol]. Proteins were resolved on precast gels (Bio-Rad) at 200 V for 30 min and stained with Coomassie dye (ProBlue Safe Stain, Giotto Biotech). Gels were imaged by using a ChemiDoc XRS system (Bio-Rad). Image analysis was performed with Image Lab software, version 5.0 (Bio-Rad). For quantification, the background intensity, calculated from the average of the areas without bands, was subtracted from each standard band intensity. Similarly, the intensity of each CA band in the transfected samples was corrected by subtracting the corresponding band intensity from the negative control at the same dilution. A calibration curve was generated in Excel (Microsoft) by plotting the intensities of the standard bands against their known concentrations. The protein concentrations of the lysate samples were then interpolated from this calibration curve based on their corrected band intensities. The final concentration of each sample was calculated as the average of the concentrations obtained from the different sample dilutions that were run on the same gel.

### In-Cell and Cell Lysate ^1^H–^15^N NMR Spectroscopy

After 48 h of expression in a ^15^N-labeled medium, cells were harvested, resuspended in 185 μL of NMR buffer (DMEM, 70 mM HEPES, and 20% (v/v) D_2_O), and transferred to a 3 mm Shigemi, following a previously established protocol.^36^ Before NMR acquisition, the suspension was gently centrifuged to form a soft pellet.

For the analysis of cell lysates, cells were recollected and lysed after the in-cell NMR experiments. Pellets were separated from the NMR buffer by centrifugation and lysed in 150 μL of phosphate-buffered saline (PBS, Life Technologies) by the freeze–thaw method. Soluble lysates were obtained by centrifugation and analyzed by ^1^H–^15^N NMR spectroscopy with 10% (v/v) of D_2_O.

^1^H–^15^N NMR spectra on cells and cell lysates were recorded at 310 K at a 950 MHz Bruker Avance NEO spectrometer equipped with a 5 mm TCI Cryoprobe. 2D ^1^H–^15^N SOFAST-HMQC spectra (Bruker pulse sequence sfhmqcf3gpph) were recorded on cell samples and on the corresponding lysates with frequency offsets of 4.7 ppm (^1^H) and 118 ppm (^15^N), spectral windows of 16 ppm (^1^H) and 50 ppm (^15^N), acquisition times of 67.4 ms (^1^H) and 13.3 ms (^15^N), and an interscan delay of 0.3 s using the shaped pulses Pc9_4_90.1000 and Reburp.1000 for selective ^1^H inversion and refocusing, respectively.

The excitation width and offset were set to 6 and 8.7 ppm, respectively. Shaped pulse lengths and power levels were automatically calculated (-DCALC_SP option in the pulse sequence). 64 initial scans and 128 increments were employed, resulting in a total experimental time of 58 min. To remove the background signals arising from the incorporation of ^15^N in other cellular components, each 2D NMR spectrum recorded on cells and lysates was further processed in Topspin (Bruker) by subtracting a spectrum recorded using identical parameters on cells transfected with an empty vector and on the corresponding lysate, as previously described.^36^

### Synthesis and Characterization of Fluorinated Compounds

Anhydrous solvents and all reagents were purchased from Merck, Fluorochem, and TCI. All reactions involving air- or moisture-sensitive compounds were performed under a nitrogen atmosphere using dried glassware and syringe techniques to transfer solutions. ^1^H NMR, ^13^C NMR, and ^19^F NMR spectra were recorded using a Bruker Advance III 400 MHz spectrometer in DMSO-*d*_6_. Chemical shifts are reported in parts per million (ppm), and the coupling constants (*J*) are expressed in Hertz (Hz). Splitting patterns are designated as follows: s, singlet; d, doublet; t, triplet; q, quadruplet; m, multiplet; and dd, doublet of doublets. The assignment of exchangeable protons was confirmed by the addition of D_2_O. Analytical thin-layer chromatography (TLC) was carried out on Sigma-Aldrich silica gel F-254 plates. Flash chromatography purifications were performed on Sigma-Aldrich Silica gel 60 (230–400 mesh ASTM) as the stationary phase, and ethyl acetate/*n*-hexane or MeOH/DCM were used as eluents. All spectra were in accord with the assigned structures. All final compounds were >95% pure by HPLC. High-resolution mass spectrometry (HRMS) analysis was performed by using an LTQ Orbitrap XL hybrid mass spectrometer (HRMS system) equipped with an Ion Max electrospray ionization (ESI) source operating in the positive ion mode at 60,000 fwhm mass resolution (referred at 400 *m*/*z*). ESI source and ion optics parameters were optimized on the protonated molecule of each analyte as follows: ESI voltage was 4 kV, capillary voltage was 13 V, tube lens voltage was 60 V, and the capillary temperature was 290 °C. Sheath and auxiliary gas were nitrogen at 15 and 8 arbitrary units, respectively. The stock solution of each analyte was prepared at 1 mg/mL in methanol containing 10% DMSO, and then, by a proper dilution, the respective working solution at 20 mg/mL in acetonitrile (ACN)/water 1:1 containing 0.1% formic acid (FA) was prepared. The accurate *m*/*z* ratio measurement was carried out by introducing into the HRMS system via a syringe pump at 10 μL/min the working solution of each analyte, monitoring the *m*/*z* range between 160 and 1000. The purity of target compounds was assessed by HPLC using an Agilent 1200 Series gradient HPLC system with a Luna PFP column (3 μm, 2 mm × 30 mm) at a flow rate of 0.25 mL/min and a linear gradient of the mobile phase, i.e., 10 mM FA and 5 mM ammonium formate in ultrapure water solution (solvent A) and 10 mM FA and 5 mM ammonium formate in ACN (solvent B). Representative HPLC traces are shown in Figure S9.

The synthesis of 4-(3-(4-(trifluoromethyl)­phenyl)­ureido)­benzenesulfonamide (**4**) was carried out as described by Pacchiano et al. (referred to as compound **11**).^30^

### Synthesis of Compounds 2,2,2-Trifluoro-*N*-(4-sulfamoylphenyl)­acetamide (**1**) and 2,2,2-Trifluoro-*N*-(4-sulfamoylphenethyl)­acetamide (**3**) (Scheme 1)

Compounds **1** and **3** were synthesized using a similar synthetic approach to that employed previously for compound **1** by Scozzafava et al.^29^ Trifluoroacetic anhydride (1.2 equiv) was carefully added at 0 °C to a solution of sulfanilamide or 4-sulfamoylphenethylamine (0.2 g, 1 equiv) in anhydrous pyridine (5 mL) under a nitrogen atmosphere. The reaction mixture was warmed at rt and stirred for 0.5 h. After monitoring by TLC (EtOAc/hexane), slush and HCl 12 M were added until pH = 4, and the resulting solution was extracted in EtOAc (25 mL × 3). The collected organic phases were dried with Na_2_SO_4_, filtered, and evaporated to yield a yellow oil. It was purified by a silica gel chromatography column (EtOAc/hexane), achieving **1** and **3** as white powders.

### 2,2,2-Trifluoro-*N*-(4-sulfamoylphenyl)­acetamide (**1**)

Yield 45%; silica gel TLC *R_f_* 0.54 (MeOH/DCM 6% v/v); δ_H_ (400 MHz, DMSO-*d*_6_): 11.59 (s, 1H, exchange with D_2_O, CON*H*), 7.89 (s, 4H, 4 × Ar–*H*), 7.40 (s, 2H, exchange with D_2_O, SO_2_N*H*_2_); δ_C_ (100 MHz, DMSO-*d*_6_): 156.4 (q,^2^ *J*_C–F_ = 41.9 Hz), 141.9, 140.2, 127.8, 122.0, 120.9 (q,^1^ *J*_C–F_ = 287.2 Hz); δ_F_ (376 MHz, DMSO-*d*_6_): −73.93; HRMS (*m*/*z*): calcd for C_8_H_7_F_3_N_2_O_3_S ([M – H]^+^), 268.2102, found: 268.2109.

### 2,2,2-Trifluoro-*N*-(4-sulfamoylphenethyl)­acetamide (**3**)

Yield 45%; silica gel TLC *R_f_* 0.54 (MeOH/DCM 6% v/v); δ_H_ (400 MHz, DMSO-*d*_6_): 9.55 (bs, 1H, exchange with D_2_O, CON*H*), 7.79 (d, *J* = 7.9 Hz, 2H, 2 × Ar–*H*), 7.44 (d, *J* = 7.9 Hz, 2H, 2 × Ar–*H*), 7.30 (bs, 2H, exchange with D_2_O, SO_2_N*H*_2_), 3.94 (t, *J* = 6.1 Hz, 2H, C*H*_2_), 2.92 (t, *J* = 6.1 Hz, 2H, C*H*_2_); δ_C_ (100 MHz, DMSO-*d*_6_): 157.8 (q,^2^ *J*_C–F_ = 39.9 Hz), 143.8, 143.0, 130.1, 126.7, 121.3 (q,^1^ *J*_C–F_ = 289.3 Hz), 34.8; δ_F_ (376 MHz, DMSO-*d*_6_): −74.38; HRMS (*m*/*z*): calcd for C_10_H_11_F_3_N_2_O_3_S ([M – H]^+^), 296.2642, found: 296.2630.

### Synthesis of 2,2,2-Trifluoro-*N*-(4-sulfamoylbenzyl)­acetamide (**2**) (Scheme 2)

Et_3_N (1.2 equiv) and trifluoroacetic anhydride (1.2 equiv) were carefully added at 0 °C to a solution of 4-sulfamoylbenzylamine (0.2 g, 1 equiv) in anhydrous pyridine (5 mL) under a nitrogen atmosphere. The reaction mixture was warmed at rt and stirred for 0.5h. After monitoring by TLC (EtOAc/hexane), slush and HCl 12 M were added until pH = 4, and the resulting solution was extracted in EtOAc (25 mL × 3). The collected organic phases were dried with Na_2_SO_4_, filtered, and evaporated to yield a yellow oil. It was purified by a silica gel chromatography column (EtOAc/hexane), achieving **2** as a white powder.

Yield 45%; silica gel TLC *R_f_* 0.54 (MeOH/DCM 6% v/v); δ_H_ (400 MHz, DMSO-*d*_6_): 10.12 (s, 1H, exchange with D_2_O, CON*H*), 7.85 (d, *J* = 7.1 Hz, 2H, 2 × Ar–*H*), 7.48 (d, *J* = 7.1 Hz, 2H, 2 × Ar–*H*), 7.38 (s, 2H, exchange with D_2_O, SO_2_N*H*_2_), 4.50 (s, 2H, C*H*_2_); δ_C_ (100 MHz, DMSO-*d*_6_): 158.1 (q,^2^ *J*_C–F_ = 42.9 Hz), 144.2, 142.4, 128.8, 126.9, 121.3 (q,^1^ *J*_C–F_ = 287.1 Hz), 43.3; δ_F_ (376 MHz, DMSO-*d*_6_): −74.27; HRMS (*m*/*z*): calcd for C_9_H_9_F_3_N_2_O_3_S ([M – H]^+^), 282.2372, found: 282.2380.

### Synthesis of 4-(3-(4-(Trifluoromethoxy)­phenyl)­ureido)­benzenesulfonamide (**5**) (Scheme 3)

Sulfanilamide (0.2 g, 1 equiv) and DIPEA (0.05 equiv) were added at rt to a solution of 4-trifluoromethoxyphenyl isocyanate (1.1 equiv) in anhydrous ACN under a nitrogen atmosphere. The reaction mixture was then stirred on. After monitoring by TLC (MeOH/DCM), the resulting precipitate was filtered and washed with cold ACN, achieving **5** as a white powder.

Yield 45%; silica gel TLC *R_f_* 0.54 (MeOH/DCM 6% v/v); δ_H_ (400 MHz, DMSO-*d*_6_): 9.14 (s, 1H, exchange with D_2_O, NHCON*H*), 9.03 (s, 1H, exchange with D_2_O, N*H*CONH), 7.77 (d, *J* = 9.1 Hz, 2H, 2 × Ar–*H*), 7.64 (d, *J* = 8.7 Hz, 2H, 2 × Ar–*H*), 7.60 (d, *J* = 9.1 Hz, 2H, 2 × Ar–*H*), 7.34 (d, *J* = 8.7 Hz, 2H, 2 × Ar–*H*), 7.24 (s, 2H, exchange with D_2_O, SO_2_N*H*_2_); δ_C_ (100 MHz, DMSO-*d*_6_): 153.3, 143.9, 143.7, 139.6, 138.1, 127.8, 125.1 (q,^1^ *J*_C–F_ = 255.8 Hz), 122.7, 120.7, 118.6; δ_F_ (376 MHz, DMSO-*d*_6_): −57.07; HRMS (*m*/*z*): calcd for C_14_H_12_F_3_N_3_O_4_S ([M – H]^+^), 375.3222, found: 375.3229.

### Synthesis of 4-((2,2,2-Trifluoroethoxy)­methyl)­benzenesulfonamide (**6**) and 4-((3,3,3-Trifluoro-2,2-bis­(trifluoromethyl)­propoxy)­methyl)­benzenesulfonamide (**7**) (Scheme 4)

#### Step I

NH_4_OH 28–30% (2 equiv) was slowly added at 0 °C to a solution of 4-bromomethylbenzenesulfonyl chloride (0.3 g, 1 equiv) in THF, and then the reaction mixture was warmed at rt and stirred for 2 h. After monitoring by TLC (EtOAC/hexane), the solvent was evaporated, and the resulting crude product was suspended in H_2_O. HCl (12M) was added until pH = 4, and it was filtered and washed with H_2_O and Et_2_O, obtaining **8** as a white powder. It was used in the next step without further purification.

Yield 95%; silica gel TLC *R_f_* 0.46 (EtOAc/hexane 50% v/v); δ_H_ (400 MHz, DMSO-*d*_6_): 7.84 (d, *J* = 8.3 Hz, 2H, 2 × Ar–*H*), 7.67 (d, *J* = 8.3 Hz, 2H, 2 × Ar–*H*), 7.42 (s, 2H, exchange with D_2_O, SO_2_N*H*_2_), 4.80 (s, 2H, C*H*_2_); δ_C_ (100 MHz, DMSO-*d*_6_): 144.7, 142.8, 130.7, 127.0, 33.9.^37^

#### Step II

DMF dimethyl acetal (1.2 equiv) was carefully added at 0 °C to a solution of **8** (0.3 g, 1 equiv) in DMF (1 mL), and then the reaction mixture was warmed to rt and stirred for 0.25 h. After monitoring by TLC (EtOAc/hexane), slush was added, and the resulting suspension was filtered, achieving **9** as a white powder. It was used in the next step without further purification.

Yield 78%; silica gel TLC *R_f_* 0.18 (EtOAc/hexane 50% v/v); δ_H_ (400 MHz, DMSO-*d*_6_): 8.26 (s, 1H, CH), 7.80 (d, *J* = 8.3 Hz, 2H, 2 × Ar–*H*), 7.63 (d, *J* = 8.3 Hz, 2H, 2 × Ar–*H*), 4.79 (s, 2H, C*H*_2_), 3.18 (s, 3H, C*H*_3_), 2.95 (s, 3H, C*H*_3_).

#### Step III

NaH (1.5 equiv) was carefully added at 0 °C to a solution of 2,2,2-trifluoroethanol or 3,3,3-trifluoro-2,2-bis­(trifluoromethyl)­propan-1-ol (1.5 equiv) in anhydrous DMF (2 mL) under a nitrogen atmosphere, then the resulting suspension was stirred for 0.5 h at 0 °C. After that, **9** (0.3 g, 1 equiv) was added, and the reaction mixture was stirred at rt. After monitoring by TLC (EtOAc/hexane), slush was added, and it was extracted in EtOAc (25 mL × 3). The collected organic phases were washed with brine (15 mL × 3) and dried with Na_2_SO_4_, and then it was filtered and evaporated, achieving **10** or **11** as yellow powders. It was used in the next step without further purification.

### (*E*)-*N*,*N*-Dimethyl-*N*′-((4-((2,2,2-trifluoroethoxy)­methyl)­phenyl)­sulfonyl)­formimidamide (**10**)

Yield 81%; silica gel TLC *R_f_* 0.21 (EtOAc/hexane 50% v/v); δ_H_ (400 MHz, DMSO-*d*_6_): 8.25 (s, 1H, CH), 7.81 (d, *J* = 8.1 Hz, 2H, 2 × Ar–*H*), 7.52 (d, *J* = 8.1 Hz, 2H, 2 × Ar–*H*), 4.76 (s, 2H, C*H*_2_), 4.18 (m, 2H, C*H*_2_), 3.18 (s, 3H, C*H*_3_), 2.94 (s, 3H, C*H*_3_).

### (*E*)-*N*,*N*-Dimethyl-N′-((4-((3,3,3-trifluoro-2,2-bis­(trifluoromethyl)­propoxy)­methyl)­phenyl)­sulfonyl)­formimidamide (**11**)

Yield 63%; silica gel TLC *R_f_* 0.27 (EtOAc/hexane 50% v/v); δ_H_ (400 MHz, DMSO-*d*_6_): 8.26 (s, 1H, CH), 7.80 (d, *J* = 7.9 Hz, 2H, 2 × Ar–*H*), 7.62 (d, *J* = 7.9 Hz, 2H, 2 × Ar–*H*), 4.78 (s, 2H, C*H*_2_), 3.18 (s, 3H, C*H*_3_), 2.94 (s, 3H, C*H*_3_).

#### Step IV

Hydrazine hydrate (10 equiv) was carefully added at rt to a solution of **10** or **11** (0.27 g, 1 equiv) in EtOH (5 mL). The reaction mixture was stirred for 1 h. After monitoring by TLC (EtOAc/hexane), the solvent was evaporated and the resulting crude was suspended in cold H_2_O, and then it was filtered and purified by silica gel chromatography column (EtOAc/hexane), achieving **6** or **7** as white powders.

### 4-((2,2,2-Trifluoroethoxy)­methyl)­be5nzenesulfonamide (**6**)

Yield 53%; silica gel TLC *R_f_* 0.42 (EtOAc/hexane 80% v/v); δ_H_ (400 MHz, DMSO-*d*_6_): 7.86 (d, *J* = 7.4 Hz, 2H, 2 × Ar–*H*), 7.56 (d, *J* = 7.4 Hz, 2H, 2 × Ar–*H*), 7.41 (s, 2H, exchange with D_2_O, SO_2_N*H*_2_), 4.78 (s, 2H, C*H*_2_), 4.19 (m, 2H, C*H*_2_); δ_C_ (100 MHz, DMSO-*d*_6_): 144.6, 142.1, 129.6 (q,^1^ *J*_C–F_ = 276.8 Hz), 128.8, 126.8, 73.4, 68.4 (q,^2^ *J*_C–F_ = 32.7 Hz); δ_F_ (376 MHz, DMSO-*d*_6_): −72.76; HRMS (*m*/*z*): calcd for C_9_H_10_F_3_NO_3_S ([M – H]^+^), 269.2382, found: 269.2371.

### 4-((3,3,3-Trifluoro-2,2-bis­(trifluoromethyl)­propoxy)­methyl)­benzenesulfonamide (**7**)

Yield 45%; silica gel TLC *R_f_* 0.54 (MeOH/DCM 6% v/v); δ_H_ (400 MHz, DMSO-*d*_6_): 7.91 (d, *J* = 8.1 Hz, 2H, 2 × Ar–*H*), 7.63 (d, *J* = 8.1 Hz, 2H, 2 × Ar–*H*), 7.45 (s, 2H, exchange with D_2_O, SO_2_N*H*_2_), 5.28 (s, 2H, C*H*_2_); δ_C_ (100 MHz, DMSO-*d*_6_): 145.5, 139.4, 129.3, 127.0, 125.4 (q,^1^ *J*_C–F_ = 294.1 Hz), 81.0 (q,^2^ *J*_C–F_ = 31.1 Hz), 71.8; δ_F_ (376 MHz, DMSO-*d*_6_): −69.77; HRMS (*m*/*z*): calcd for C_12_H_10_F_9_NO_3_S ([M – H]^+^), 419.2616, found: 419.2608.

### CA Inhibition Assay

An Applied Photophysics stopped-flow instrument has been used for assaying the CA-catalyzed CO_2_ hydration activity.^38^ Phenol red (at a concentration of 0.2 mM) has been used as an indicator, working at the absorbance maximum of 557 nm, with 20 mM HEPES (pH 7.5) as buffer, and 20 mM Na_2_SO_4_ (to maintain a constant ionic strength), following the initial rates of the CA-catalyzed CO_2_ hydration reaction for a period of 10–100 s. The CO_2_ concentrations ranged from 1.7 to 17 mM for the determination of the kinetic parameters and inhibition constants. For each inhibitor, at least six traces of the initial 5–10% of the reaction have been used for determining the initial velocity. The uncatalyzed rates were determined in the same manner and subtracted from the total observed rates. Stock solutions of inhibitor (0.1 mM) were prepared in distilled–deionized water, and dilutions up to 0.01 nM were done thereafter with the assay buffer. Inhibitor and enzyme solutions were preincubated together for 1 h at room temperature prior to assay, in order to allow for the formation of the complex. The inhibition constants were obtained by nonlinear least-squares methods using PRISM 3 and the Cheng-Prusoff equation, as reported earlier,^39,40^ and represent the mean from at least three different determinations. All CA isoforms were recombinant ones obtained in-house as reported earlier.^41^

### Ligand Stability Assessed by ^19^F NMR Spectroscopy

Fluorinated compounds **1**–**7** were dissolved in DMSO at a final concentration of 80 mM. To assess their stability in cell culture medium, an aliquot of each compound was diluted in DMEM supplemented with 2% fetal bovine serum (FBS), 1% penicillin/streptomycin, and 3% D_2_O, yielding a final concentration of 100 μM. Each fluorinated compound in DMEM was analyzed by ^19^F NMR spectroscopy at 37 °C, immediately after preparation and again after 1 h of incubation at 37 °C.

^19^F NMR spectra were acquired at 310 K at a 600 MHz Bruker Avance NEO spectrometer equipped with a QCI-F 5 mm Cryoprobe operating at 564.6 MHz. A single 90° pulse was used (zg Bruker pulse program), followed by 1.2 s of acquisition, with a frequency offset of −66.0 ppm and a spectral width of 49.2 ppm. Each spectrum was recorded with 32 scans and a 10 s interscan delay, resulting in a total acquisition time of approximately 7 min per sample.

### In-Cell and Cell Lysate ^19^F NMR Spectroscopy

To perform the in-cell ^19^F NMR experiments, after 48 h of protein expression in an unlabeled medium, cells were treated with 100 μM of one of the seven fluorinated ligands. For competition binding experiments, cells were incubated with two ligands simultaneously, each at a concentration of 50 μM. Incubation was carried out for 1 h at 37 °C with 5% CO_2_. Following incubation, cells were then harvested, resuspended in 185 μL of NMR buffer (DMEM, 70 mM HEPES, and 20% (v/v) D_2_O), transferred to a 3 mm Shigemi tube, and gently centrifuged to form a soft pellet. Following NMR analysis, the cell pellet was resuspended, collected in a tube, and centrifugated. The supernatant was collected and analyzed by ^19^F NMR spectroscopy, while the cell pellet was lysed in 150 μL of PBS. The resulting soluble lysate was subsequently analyzed by ^19^F NMR spectroscopy with 10% (v/v) of D_2_O.

^19^F NMR spectra were recorded at 310 K at a 600 MHz Bruker Avance III equipped with a room-temperature SEL-HP probe tuned at 564.6 MHz for ^19^F detection. The ^19^F chemical shift scale was referenced to trichlorofluoromethane by setting the signal of trifluoroacetic acid in an external reference sample to −76.55 ppm. A single 90° pulse was employed, followed by 0.3 s acquisition with a frequency offset of −54.3 ppm and a spectral window of 50.3 ppm. Cell samples and the corresponding lysates and supernatants, a set of four spectra with 1280 scans each and an interscan delay of 1 s were recorded on each sample for a total acquisition time of 112 min. The spectra were processed in Topspin with 10 and 5 Hz exponential line broadening for cells and lysates, respectively, phase-corrected, and summed together. A polynomial baseline correction was applied to the sum spectrum to remove a strong baseline distortion arising from poly­(tetrafluoroethylene) (PTFE) components inside the probe.

### Uptake Kinetics Assessed by ^19^F NMR Bioreactor Experiments

Transfected cells were encapsulated in agarose threads, composed of 1.5% (w/v) low-gelling agarose (Sigma-Aldrich) dissolved in PBS, following a previously described protocol.^32^ Briefly, cells from one 75 cm^2^ flask were prewarmed to 37 °C and mixed with 400 μL of agarose solution at the same temperature. The suspension was drawn into PEEK tubing (i.d. 0.75 mm) using a syringe, allowed to cool at room temperature for 2 min, and then loaded into the NMR bioreactor prefilled with PBS.

The bioreactor setup was assembled as previously reported.^32^ Briefly, gel-encapsulated cells were maintained at 37 °C in an NMR flow unit (InsightMR, Bruker) using a circulating water bath (Julabo) for temperature control. A PEEK capillary (i.d. 0.5 mm) delivered medium to the bottom of the NMR tube, while a PTFE outlet at the top ensured continuous flow. Unlabeled DMEM supplemented with 2% FBS, 10 mM NaHCO_3_, 1% antibiotics, 3% D_2_O, and the fluorinated compound was pumped at a constant flow rate of 0.1 mL/min using a peristaltic pump (Reglo ICC, Ismatec) through a temperature-controlled circuit.

For time-resolved in-cell NMR experiments performed in the bioreactor, a series of ^19^F NMR spectra was acquired at 310 K on a 600 MHz Bruker Avance NEO spectrometer equipped with a QCI-F 5 mm Cryoprobe operating at 564.6 MHz. Each spectrum was recorded with 256 scans and a 1 s interscan delay, yielding a time resolution of 5 min per spectrum over a total acquisition time of 1 h. The resulting time-series data were analyzed using the Dynamics Center (Bruker) to quantify peak areas as a function of time. The rate of uptake for each compound was obtained by linear regression of the first 7 time points of the binding curve using Origin.

### Cellular Thermal Shift Assay

For the CETSA assay, cultured cells from a T75 flask (approximately 30 million cells are required to establish one melting curve) were treated with 25 μL of DMSO stock of fluorinated compound (final compound concentration of 100 μM, DMSO concentration of 0.00125%) or with 25 μL of DMSO as the control for 1 h at 37 °C and 5% CO_2_. Cells were harvested with Accutase (Sigma-Aldrich), washed once with Hanks’ balanced salt solution (HBSS, Thermo Fisher Scientific), and resuspended in 800 μL of HBSS. The cell suspension was divided into 50 μL aliquots in PCR 0.2 mL tubes and heated at different temperatures (42.7–44.5–45.7–47.5–50.6–52.0–55.0–58.0–61.0–62.6–65.8–67.7–68.8–70.7 °C) for 3 min in a PCR machine (MyCycler Thermal Cycler, Bio-Rad) followed by a cooling at 4 °C. 50 μL of lysis buffer (HEPES 40 mM, pH 7.5, NaCl 150 mM) was added to each heat-treated aliquot, which were then frozen–thawed three times using liquid nitrogen and a heating block set at 25 °C. The resulting cell lysates were centrifuged at 14,000 × *g* for 1 h at 4 °C. Each supernatant was transferred to a new microtube and analyzed by Western blot. Samples were run on SDS-PAGE as described above and subsequently transferred to a 0.2 μm nitrocellulose membrane (Trans-Blot Turbo Transfer Pack, Bio-Rad) using the Bio-Rad transfer System. The membrane was blocked for 1 h at room temperature in blocking buffer [5.0% low-fat dry milk in tris-buffered saline (TBS) with 0.1% Tween-20], washed three times with TBS containing 0.1% Tween-20 (TBST). The primary rabbit anti-CA2 antibody (Proteintech 16961–1-AP; Lot #00082518) was diluted 1:2000 in a buffer prepared with 2.0% (w/v) low-fat dry milk in TBST and incubated overnight at 4 °C. After washing in TBST, the membrane was incubated with goat antirabbit IgG (whole molecule)-peroxidase secondary antibody (Sigma: A0545) diluted 1:70000 for 1 h at room temperature, followed by three washes with TBST. LiteAblot EXTEND chemiluminescence substrate (EuroClone) was used for detection. Images were acquired using the ChemiDoc XRS imaging system (Bio-Rad) and quantified using Image Lab software version 5.0 (Bio-Rad). Band intensities were normalized such that the maximum intensity was set to 100% and the minimum to 0%, generating relative intensity values for each temperature point.

To construct the thermal stability curve, the normalized intensity data were plotted against the temperature and analyzed using GraphPad Prism. Nonlinear regression was applied using the four-parameter logistic (4PL) sigmoidal model, selecting the ″[Agonist] vs response – Variable slope″ function. In this model, the temperature serves as the X-variable, and the relative band intensity serves as the Y-response. Curve fitting was performed to estimate the inflection point, which corresponds to the apparent melting temperature (*T*_m_), defined as the temperature at which 50% of the protein is denatured or insoluble.

### HPLC-MS Analysis

A solution of compound **1** in Milli-Q water (100 μM) was incubated at 37 °C for 7 days and subsequently analyzed by HPLC-MS^3^. The resulting MS^3^ spectra were compared with those of reference samples of compound **1** and sulfanilamide. The analyses were performed using an HPLC-MS instrument composed of an HPLC Accela, with autosampler and column oven, coupled to an LTQ XL linear quadrupole ion trap mass spectrometer equipped with an IonMax ESI interface (Thermo Scientific). Chromatographic separation was done using a Gemini C18 column (100 × 2 mm, 5 μm, Phenomenex) using the gradient elution program reported in Table S1. Mobile phase A was LC-MS grade water, and B was LC-MS grade ACN, both containing 0.1% FA. The column was maintained at 25 °C. Injection volume was 20 μL. MS and MS^n^ parameters were optimized by infusion of H_2_O/ACN 50/50 0.1% FA solution of the standards of the two molecules. Data acquisition was done in positive polarity for the first 5.5 min of the chromatographic run, then the polarity switched to negative ion. Sheath gas, auxiliary gas, and sweep gas were 30, 15, and 1 (arbitrary units), respectively, while the spray voltage value was 4.7 kV in the two polarities: the ion transfer capillary was maintained at 275 °C. Capillary and tube lens voltages were 2 and 25 V in positive ion mode, and −20 and −60 V in negative ion mode. Data were acquired in full scan in MS and in MS/MS/MS mode; for sulfanilamide, the proton adduct ion at 173 *m*/*z* was isolated (isolation window 2.2), fragmented at 72 normalized collision energy and then the fragment ion at *m*/*z* 156 was isolated (isolation window 3.0) and fragmented at 96 normalized collision energy, while for compound **1** the deprotonated ion at *m*/*z* 267.1 was isolated (isolation window 2.8), fragmented at 37 normalized collision energy and then the fragment ion at *m*/*z* 197 was isolated (isolation window 3.0) and fragmented at 80 normalized collision energy. Activation Q and activation time were 0.25 and 30 ms, respectively. The mass spectrometer was calibrated following the manufacturer’s instructions before the analysis. Data acquisition and analysis were done using the Xcalibur software (Thermo, ver. 2.2).

## Supplementary Material





## Notes

Figures S1–S9: quantification of intracellular CA isoforms, stability of compounds, HPLC-MS of compound **1**, ^19^F NMR spectra of cell lysates and supernatants, ^19^F NMR spectra of control samples, representative HPLC traces; Table S1: HPLC elution program (PDF)
Molecular formula strings and inhibitory constants (CSV)

## Abbreviations Used

AAZ: acetazolamide
ACN: acetonitrile
CA: carbonic anhydrase
CETSA: cellular thermal shift assay
DMEM: Dulbecco’s modified Eagle medium
FA: formic acid
FBS: fetal bovine serum
fwhm: full width at half-maximum
HEK293T: human embryonic kidney 293T
HEPES: 4-(2-hydroxyethyl)-1-piperazineethanesulfonic acid
MeOH: methanol
PEI: polyethylenimine
SDS-PAGE: SDS-polyacrylamide gel electrophoresis
*T*_m_: melting temperature
