# Correction to “Intracellular Binding of Novel Fluorinated Compounds to Carbonic Anhydrase Isoforms Explored by In-Cell ^19^F NMR”

**DOI:** 10.1021/acs.jmedchem.5c03459

**Published:** 2025-12-15

**Authors:** Azzurra Costantino, Letizia Barbieri, Simone Giovannuzzi, Alessio Nocentini, Claudiu T. Supuran, Mindaugas Raitelaitis, Pär Nordlund, Lucia Banci, Enrico Luchinat

**Affiliations:** † CERM − Magnetic Resonance Center, 9300 University of Florence, via Luigi Sacconi 6, 50019 Sesto Fiorentino, Italy; ‡ 524266 Consorzio Interuniversitario Risonanze Magnetiche di Metallo Proteine − CIRMMP, via Luigi Sacconi 6, 50019 Sesto Fiorentino, Italy; § NEUROFARBA Department, Section of Pharmaceutical and Nutraceutical Sciences, University of Florence, via Ugo Schiff 6, 50019 Sesto Fiorentino, Italy; ∥ Department of Oncology-Pathology, 27106 Karolinska Institutet, 171 11 Stockholm, Sweden; ⊥ Chemistry Department, University of Florence, Via della Lastruccia 3, 50019 Sesto Fiorentino, Italy

There is an error in the structure of compound **7** shown in Scheme 4. The C­(CF_3_)_3_ group was incorrectly depicted as being linked via a methylene spacer to the oxygen atom, whereas it should be directly attached. The corrected structure and corresponding synthetic procedure are now provided below. These corrections do not affect the conclusions or overall findings of the paper. The authors apologize for any inconvenience caused.

**Scheme 4:**
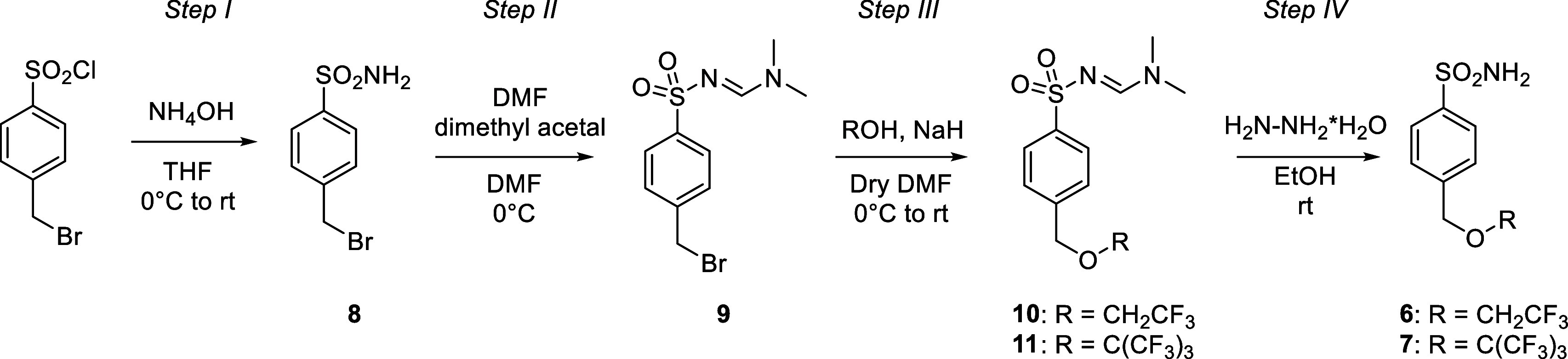
Synthesis of Compounds **6** and **7**

*Synthesis of 4-((2,2,2-Trifluoroethoxy)­methyl)­benzenesulfonamide (**6**) and 4-(((1,1,1,3,3,3-Hexafluoro-2-(trifluoromethyl)­propan-2-yl)­oxy)­methyl)­benzenesulfonamide (**7**) (Scheme 4)*.

*Step III*: NaH (1.5 equiv) was carefully added at 0 °C to a solution of 2,2,2-trifluoroethanol or 1,1,1,3,3,3-hexafluoro-2-(trifluoromethyl)­propan-2-ol (1.5 equiv) in anhydrous DMF (2 mL) under a nitrogen atmosphere, then the resulting suspension was stirred for 0.5 h at 0 °C. After that, **9** (0.3 g, 1 equiv) was added and the reaction mixture was stirred on at rt. After monitoring by TLC (EtOAc/Hexane), slush was added and it was extracted in EtOAc (25 mL × 3). The collected organic phases were washed with Brine (15 mL × 3) and dried with Na_2_SO_4_, then it was filtered and evaporated achieving **10** or **11** as yellow powders. It was used in the next step without further purification.

*(E)-N’-((4-(((1,1,1,3,3,3-Hexafluoro-2-(trifluoromethyl)­propan-2-yl)­oxy)­methyl)­phenyl)­sulfonyl)-N,N-dimethylformimidamide (**11**).* Yield 63%; silica gel TLC R*_f_* 0.27 (EtOAc/Hexane 50% v/v); δ_H_ (400 MHz, DMSO-*d*_6_): 8.26 (s, 1H, CH), 7.80 (d, *J* = 7.9 Hz, 2H, 2 × Ar-*H*), 7.62 (d, *J* = 7.9 Hz, 2H, 2 x Ar-*H*), 4.78 (s, 2H, C*H*_2_), 3.18 (s, 3H, C*H*_3_), 2.94 (s, 3H, C*H*_3_).

*4-(((1,1,1,3,3,3-Hexafluoro-2-(trifluoromethyl)­propan-2-yl)­oxy)­methyl)­benzenesulfonamide (**7**).* Yield 45%; silica gel TLC R*_f_* 0.54 (MeOH/DCM 6% v/v); δ_H_ (400 MHz, DMSO-*d*_6_): 7.91 (d, *J* = 8.1 Hz, 2H, 2 x Ar-*H*), 7.63 (d, *J* = 8.1 Hz, 2H, 2 × Ar-*H*), 7.45 (s, 2H, exchange with D_2_O, SO_2_N*H*_2_), 5.28 (s, 2H, C*H*_2_); δ_C_ (100 MHz, DMSO-*d*_6_): 145.5, 139.4, 129.3, 127.0, 125.4 (q, ^1^ *J*_C–F_ = 294.1 Hz), 81.0 (q, ^2^ *J*_C–F_ = 31.1 Hz), 71.8; δ_F_ (376 MHz, DMSO-*d*_6_): −69.77; HRMS (*m*/*z*): calcd for C_11_H_8_F_9_NO_3_S ([M – H]^−^), 405.2346, found: 405.2308.

