# Supplementary material for: Intracellular Binding of Novel Fluorinated Compounds to Carbonic Anhydrase Isoforms Explored by In-Cell 19F NMR
Source: J Med Chem. 2025 Oct 27;68(21):23363–74. doi: 10.1021/acs.jmedchem.5c02227 (PMC12621193; doi:10.1021/acs.jmedchem.5c02227)
Supplement: Supplementary file 1 [file jm5c02227_si_001.pdf]

SUPPLEMENTARY INFORMATION

Intracellular binding of novel fluorinated compounds to carbonic anhydrase isoforms explored by in-cell ^{19}F NMR

Azzurra Costantino[a], Letizia Barbieri[a,b], Simone Giovannuzzi[c], Alessio Nocentini[c] , Claudiu T. Supuran[c], Mindaugas Raitelaitis[d], Pär Nordlund[d], Lucia Banci[a,b,e], Enrico Luchinat[a,b,e,*]

[a] CERM – Magnetic Resonance Center, University of Florence, via Luigi Sacconi 6, 50019, Sesto Fiorentino, Italy;

[b] Consorzio Interuniversitario Risonanze Magnetiche di Metallo Proteine – CIRMMP, via Luigi Sacconi 6, 50019, Sesto Fiorentino, Italy;

[c] NEUROFARBA Department, Section of Pharmaceutical and Nutraceutical Sciences, University of Florence, via Ugo Schiff 6, 50019, Sesto Fiorentino, Italy;

[d] Department of Oncology-Pathology, Karolinska Institutet, 171 77 Stockholm, Sweden;

[e] Chemistry Department, University of Florence, Via della Lastruccia 3, 50019, Sesto Fiorentino, Italy.

* Email: eluchinat@cerm.unifi.it

Table of Contents **page**

Supplementary Figures S1-S9. S1

Supplementary Table S1 S11

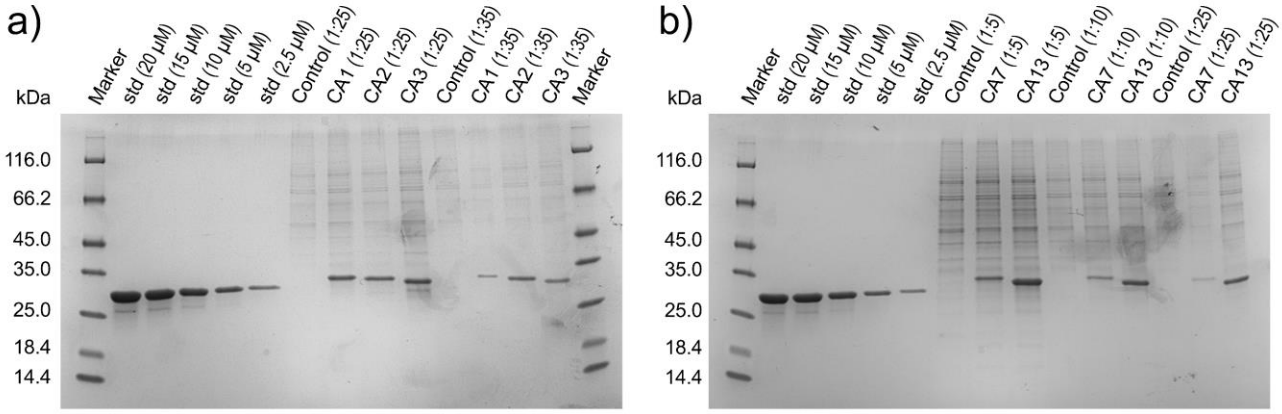

c)

Protein	Concentration (µM)		
CA1	137	±	77
CA2	130	±	11
CA3	165	±	63
CA7	47	±	16
CA13	83	±	2

Figure S1. Quantification of cytosolic carbonic anhydrase isoforms expressed in HEK293T cells. Coomassie-stained SDS-PAGE gels showing the expression levels of various cytosolic carbonic anhydrase isoforms (CA1, CA2, CA3, CA7, and CA13) in HEK293T cells. Cell lysates were obtained from T25 flasks, lysed in 150 µL of PBS, and serially diluted before gel loading. Known concentrations of purified CA2 protein were used as standards for quantification. a) SDS-PAGE showing standards and lysates expressing CA1, CA2, and CA3. b) SDS-PAGE showing standards and lysates expressing CA7 and CA13. c) Table summarizing the calculated concentrations of each cytosolic CA isoform based on band intensity analysis.

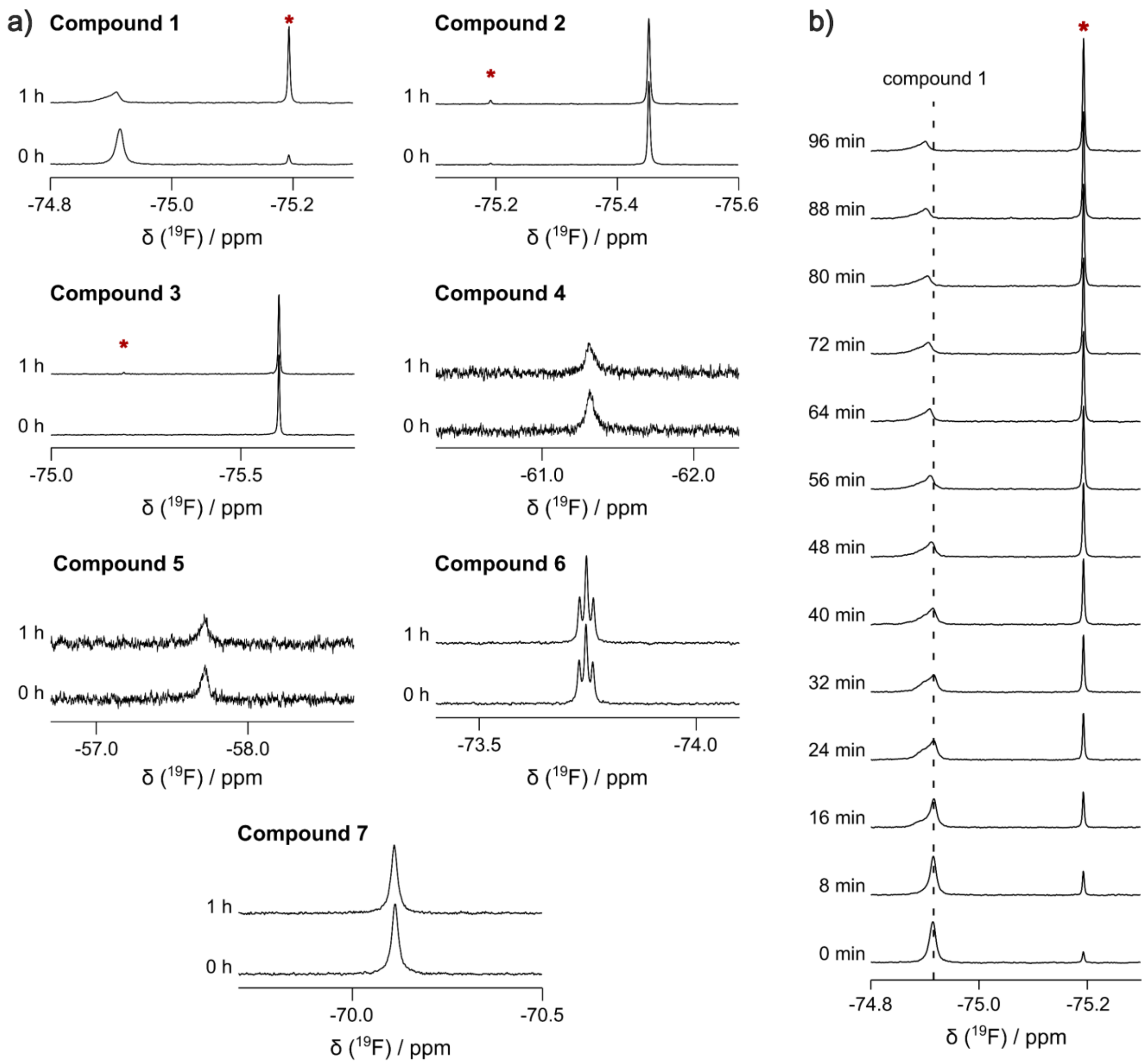

Figure S2. Stability tests of fluorinated compounds in DMEM at 37°C. a) ^{19}F NMR spectra of compounds **1**–**7** dissolved in DMEM (supplemented with 1% antibiotics, 2% FBS, and 3% D_2O), acquired at time zero and after one hour at 37°C to assess ligand stability in cell culture medium. b) A stability assay to monitor the degradation of compound **1** over time was performed by acquiring consecutive ^{19}F NMR spectra over the course of 1.5 hours. The intensities of the original compound peak and the degradation product (indicated by *) were tracked.

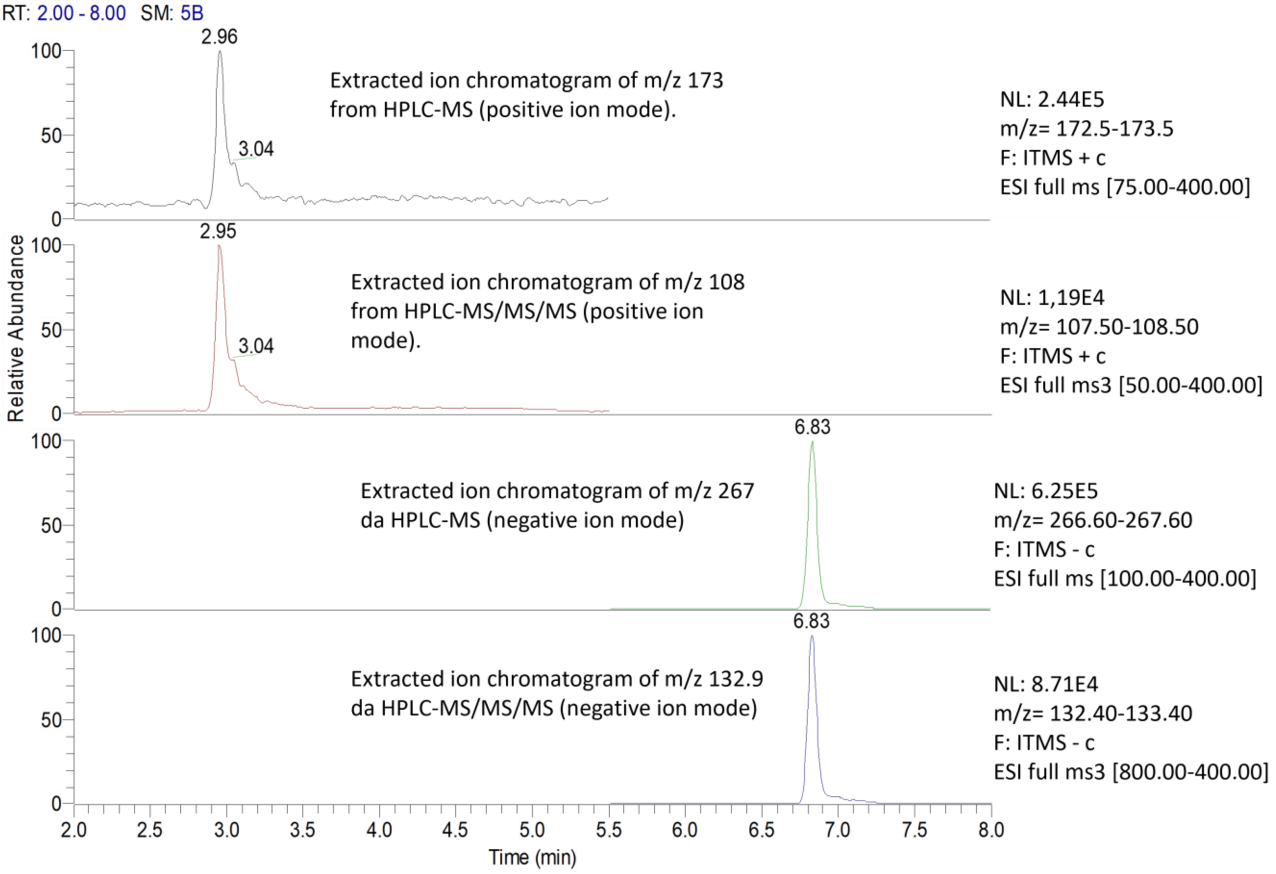

Figure S3. HPLC-MS e HPLC-MS/MS/MS of compound **1** aqueous solution. The solution contained two compounds eluting at 2.95–2.96 min and 6.83 min, respectively. The extracted ion chromatogram confirmed that the first peak corresponds to sulfanilamide, while the second peak corresponds to compound **1**, consistent with their expected molecular weights.

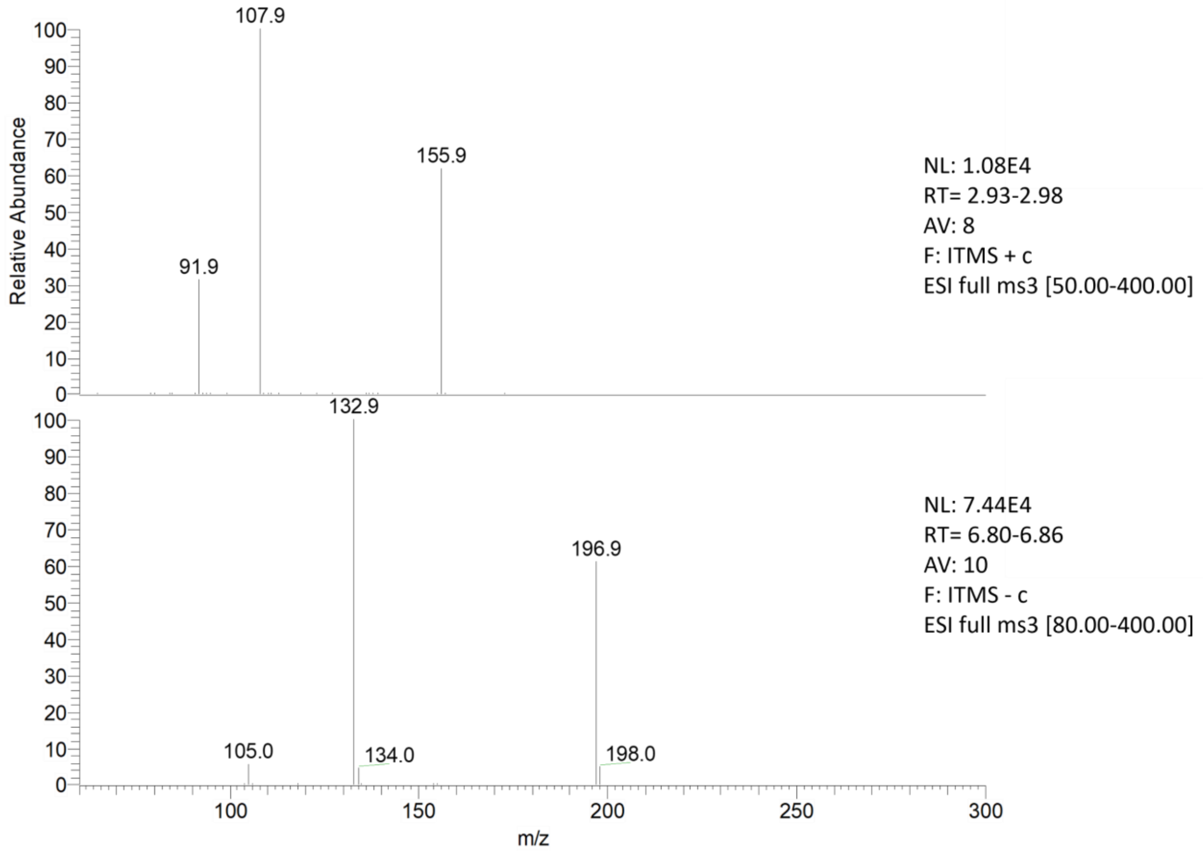

Figure S4. The ESI MS/MS/MS spectra acquired in positive ion mode (top) and negative ion mode (bottom) at the retention times of the two molecules provide clear evidence of their identities, supporting the assignment of the first peak to sulfanilamide and the second to compound **1**.

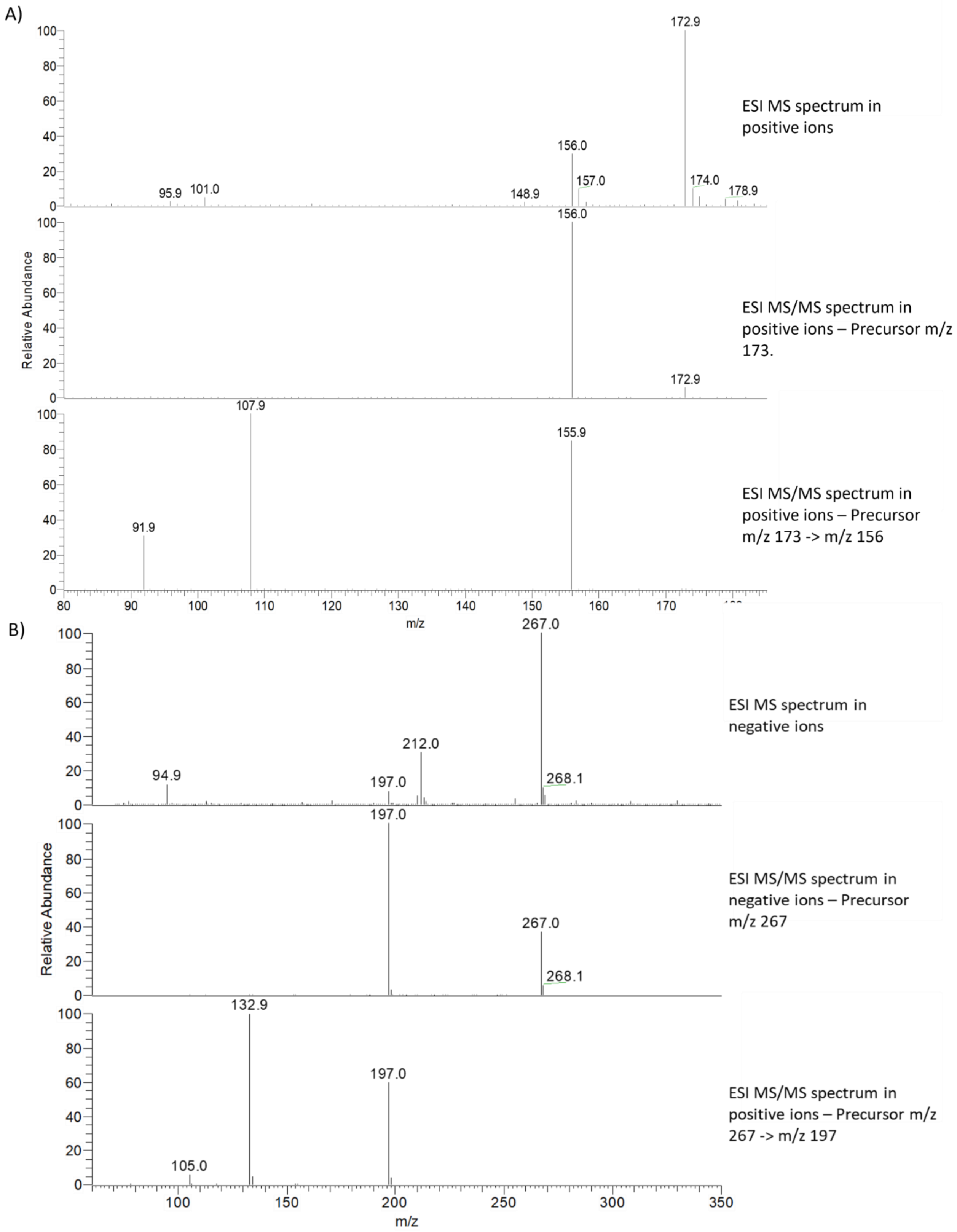

Figure S5. ESI MS, MS/MS, and MS/MS/MS spectra were recorded for A) sulfanilamide in positive ions and B) compound **1** in negative ions, serving as reference standards to identify the corresponding peaks in the analysis of the watery solution.

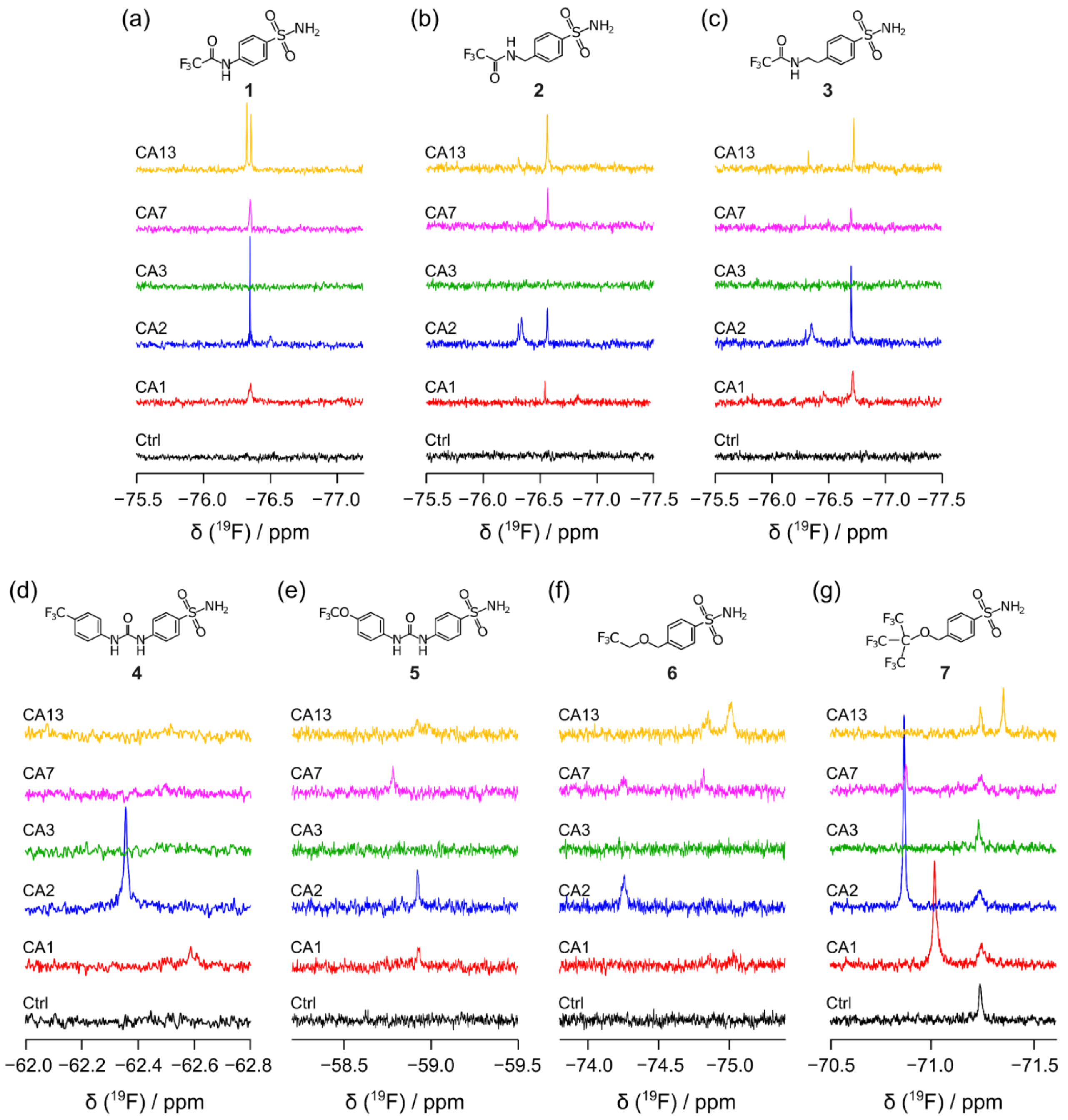

Figure S6. 1D ^{19}F NMR spectra of supernatants from cells expressing cytosolic CA isoforms and treated with fluorinated sulfonamide compounds. 1D ^{19}F NMR spectra were recorded from extracellular supernatants collected after in-cell NMR experiments on cells expressing CA1 (red), CA2 (blue), CA3 (green), CA7 (purple), and CA13 (yellow), following treatment with compounds **1** (a), **2** (b), **3** (c), **4** (d), **5** (e), **6** (f), and **7** (g). Black spectra correspond to control supernatants from cells transfected with an empty vector and treated with the same compounds.

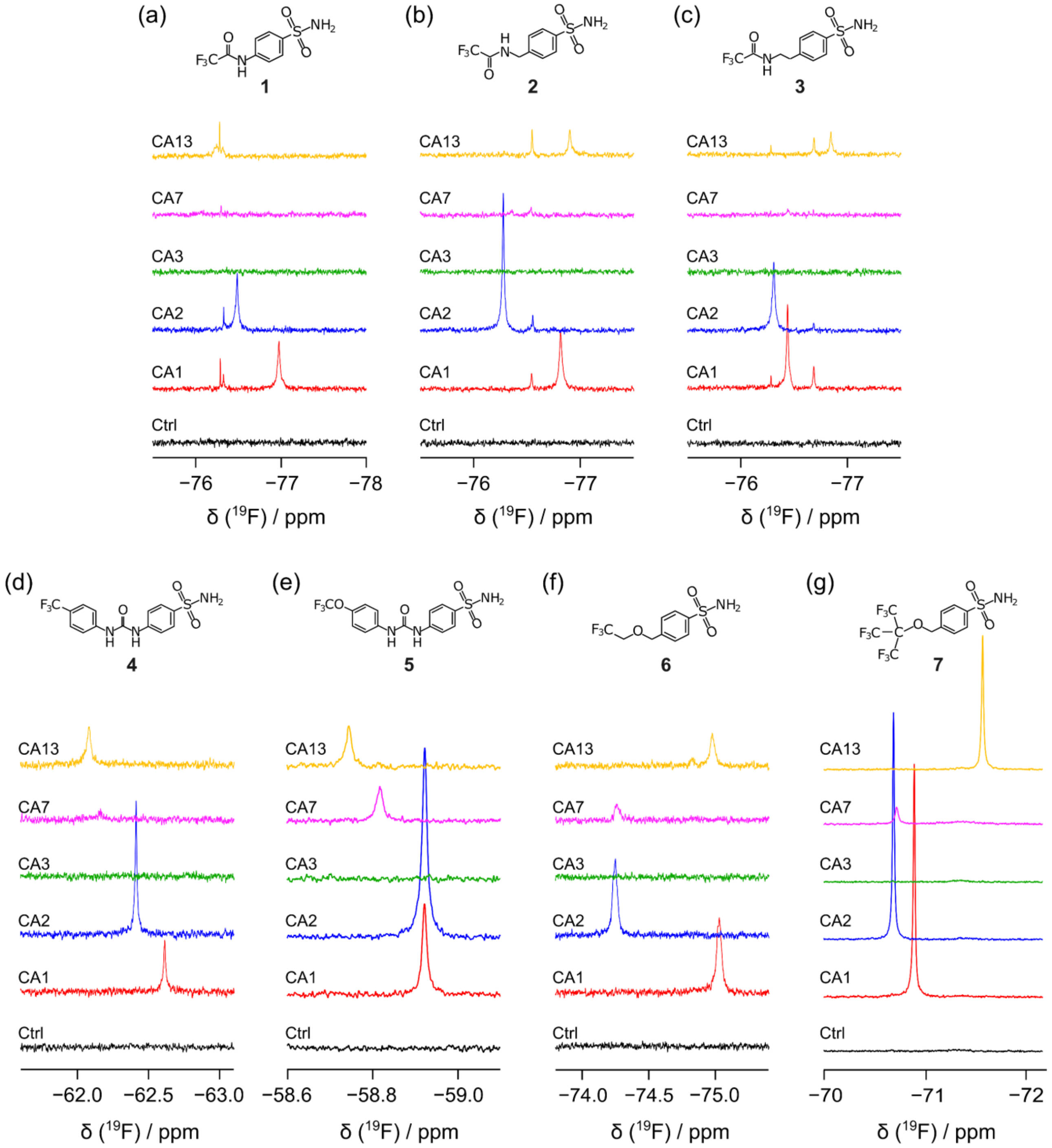

Figure S7. 1D ^{19}F NMR spectra of cell lysates from cells expressing cytosolic CA isoforms and treated with fluorinated sulfonamide compounds. Spectra were recorded from cell lysates after treatment with compound **1** (a), **2** (b), **3** (c), **4** (d), **5** (e), **6** (f), and **7** (g). Each spectrum shows the complex with the compound and a specific CA isoform: CA1 (red), CA2 (blue), CA3 (green), CA7 (purple), and CA13 (yellow). Control spectra (black) correspond to lysates from cells transfected with an empty vector and treated with the same compounds.

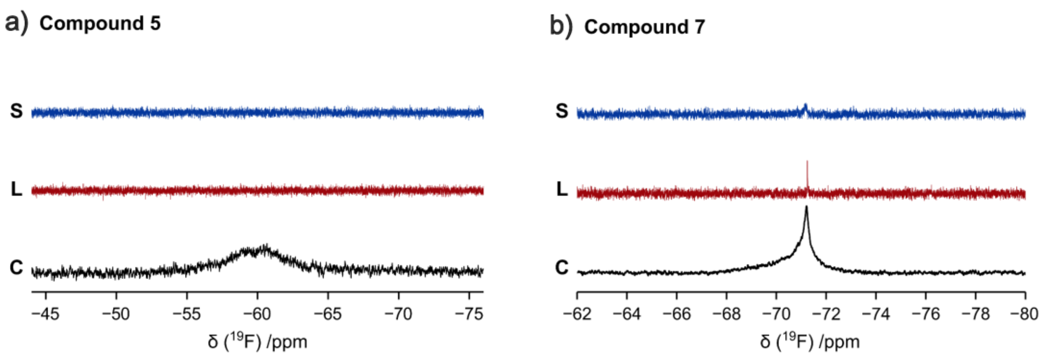

Figure S8. Detailed ^{19}F NMR spectra of in-cell, cell lysate, and supernatant samples from control cells treated with compounds **5** and **7**. 1D ^{19}F NMR spectra were acquired from HEK293T cells transfected with a vector without the gene encoding the CA isoform and treated with compound **5** (a) or compound **7** (b). Spectra are shown for intact cells (C, black), corresponding cell lysates (L, red), and extracellular supernatants collected after NMR experiments (S, blue).

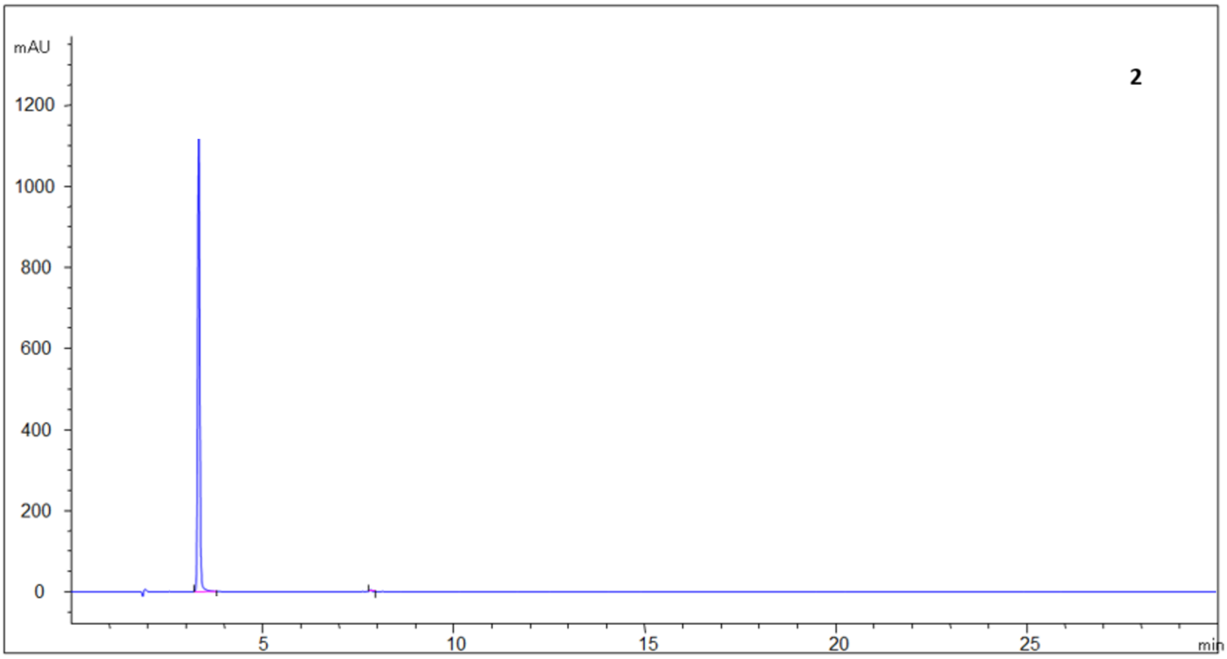

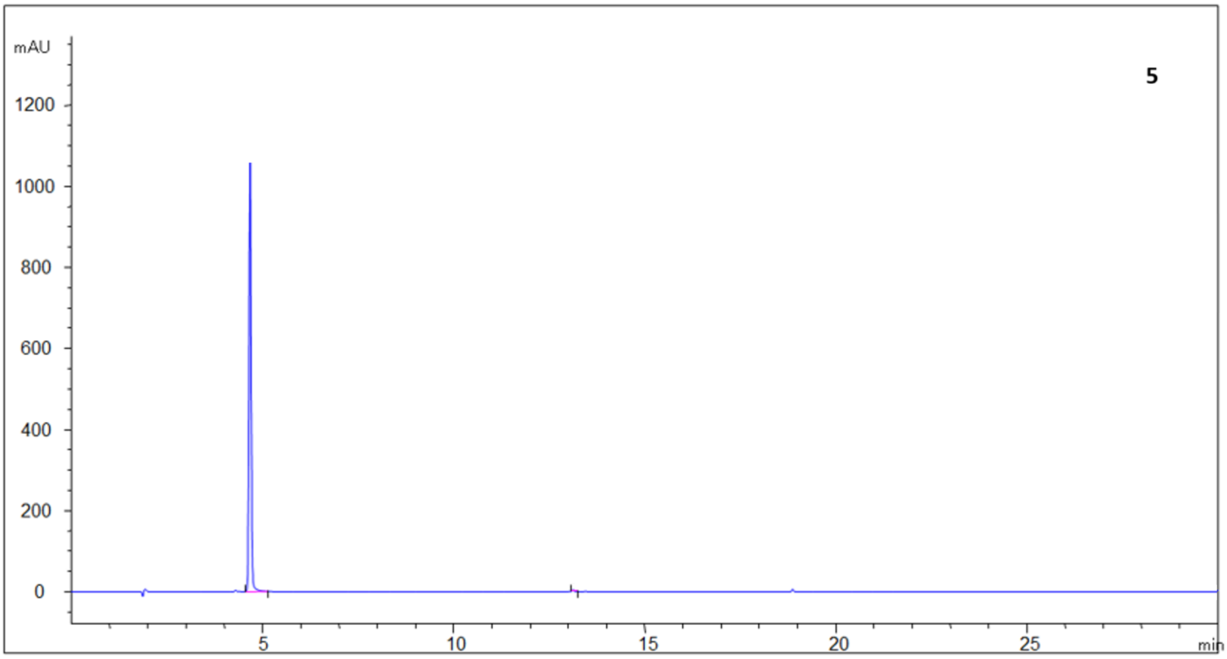

Figure S9. HPLC traces of representative compounds **2** (top) and **5** (bottom).

Time (min)	Solvent A%	Solvent B%	Flow (µL/min)
0	98	2	250
0.5	98	2	250
8	5	95	250
10	5	95	250
11	98	2	250
16	98	2	250

Table S1. HPLC gradient elution program for the chromatographic separation of compound **1** hydrolysis products. Solvent A: LC-MS grade water, 0.1% formic acid; solvent B: LC-MS grade acetonitrile, 0.1% formic acid.
